# Supplementary material for: Heart enhancers with deeply conserved regulatory activity are established early in zebrafish development
Source: Nat Commun. 2018 Nov 26;9:4977. doi: 10.1038/s41467-018-07451-z (PMC6255839; doi:10.1038/s41467-018-07451-z)
Supplement: Supplementary file 1 — Supplementary Info [file 41467_2018_7451_MOESM1_ESM.docx]

**Supplementary Informatrion**

Heart enhancers with deeply conserved regulatory activity are established early in zebrafish development

Yuan et al.


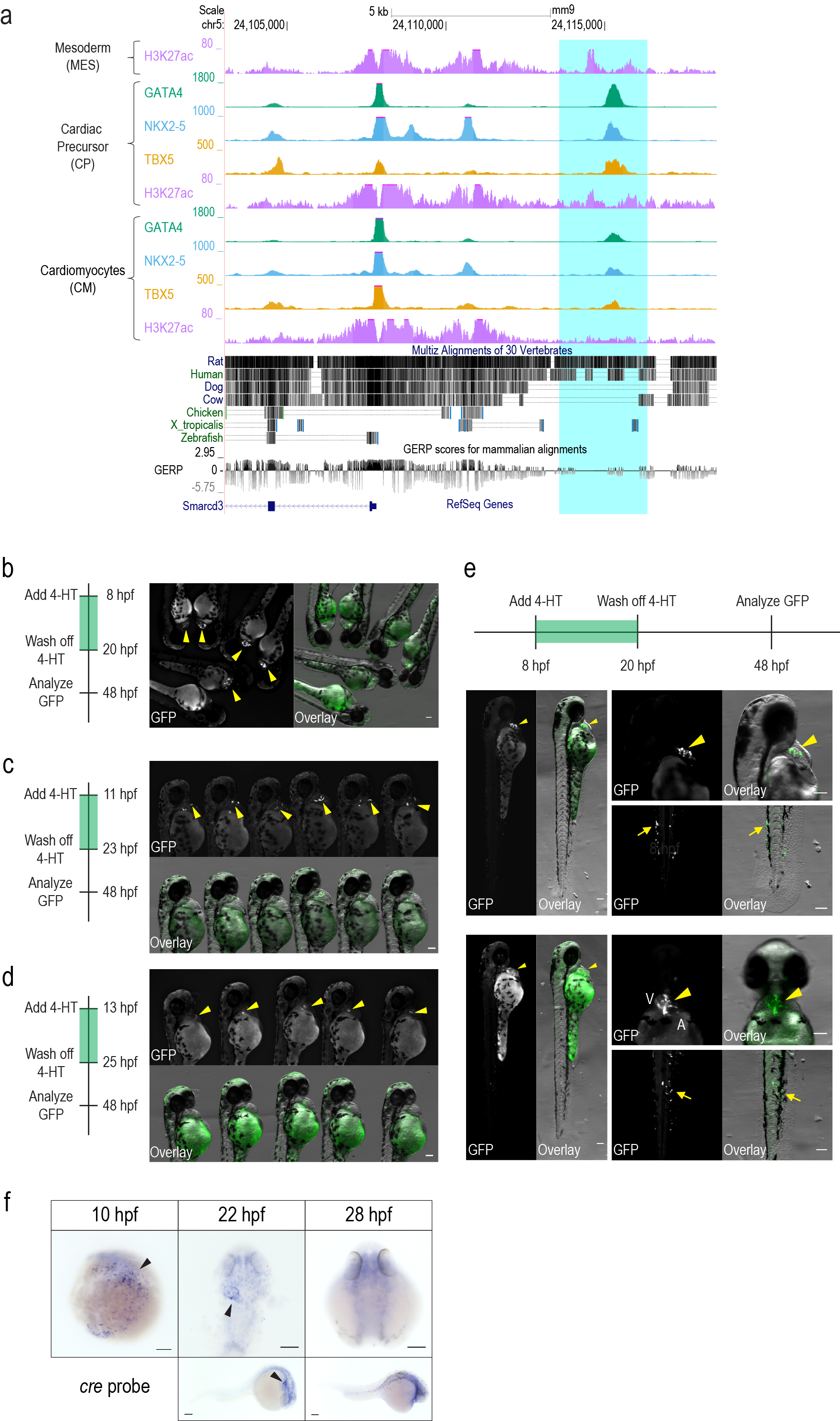


**
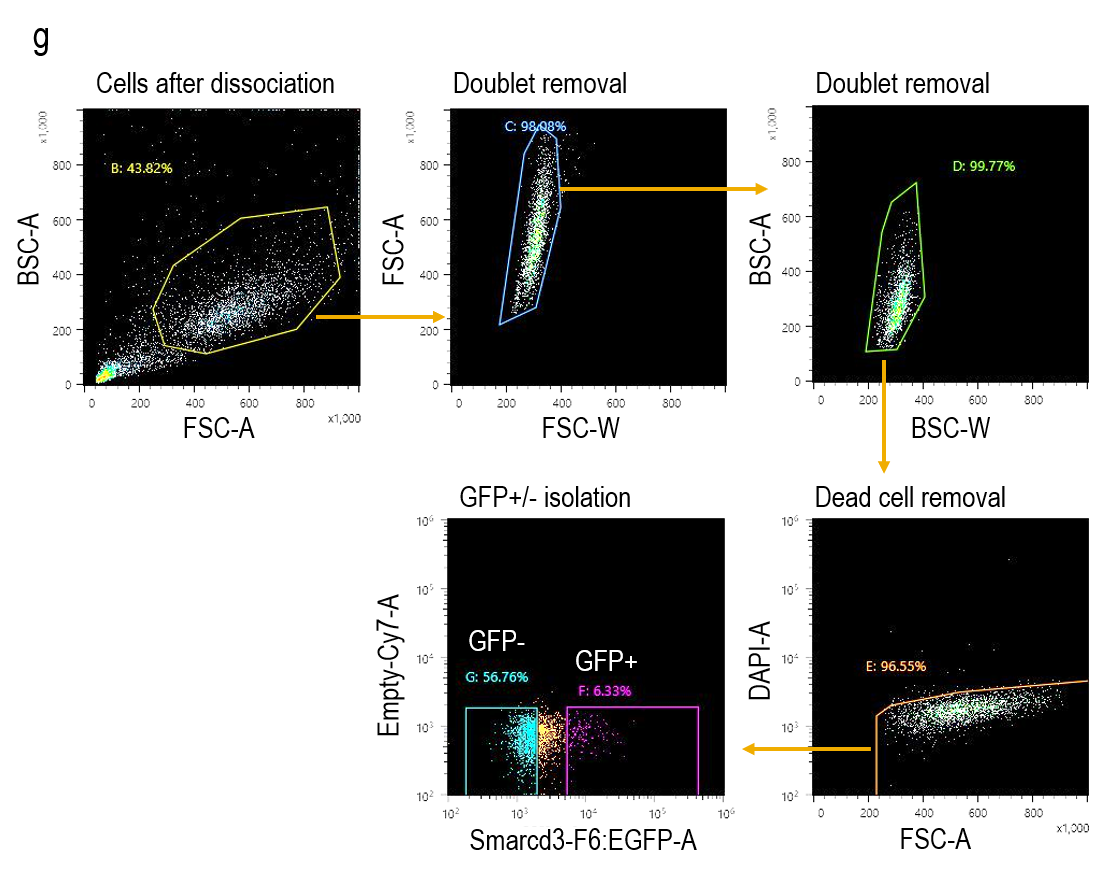
**

**Supplementary Figure 1**

**Characterization of the cardiac activity of the *Smarcd3*-F6 enhancer.**

(a) TF binding and epigenetic annotation of mouse *Smarcd3*-F6 enhancer. GATA4, NKX2.5 and TBX5 ChIP-exo average density in CP and CM^1^ and H3K27ac ChIP-seq signal intensity in MES, CP and CM^2^ were shown in this genome browser view. Cyan shaded area represents the Smarcd3-F6 enhancer region. For H3K27ac ChIP-seq, replicates from the same sample were merged and normalized to generate the intensity tracks. For TF ChIP-exo data, the average density bigwig files deposited by the authors were directly uploaded to UCSC to generate the tracks. (b-e) Lineage tracing of *Smarcd3*-F6 labeled cells. Embryos were obtained from *Tg(Smarcd3-F6:CreERT2)* and *Tg(βactin2-loxP-dsRed-loxP-GFP)* crosses. (b) (c) (d) 5 uM 4-HT was added at different time points (8 hpf, 11 hpf, 13 hpf) as indicated in each panel. GFP labeling in hearts became sparser (yellow arrow heads) as 4-HT was added at later stages, suggesting *Smarcd3*-F6 most potently marks cardiac progenitors during gastrula stages. (e) Two exemplary embryos that showed GFP switching in *Smarcd3*-F6 labeled cells when 4-HT was added at 8 hpf. GFP positive cells were most frequently observed in hearts (arrow heads) and were seen in both the ventricle and atrium (V: ventricle, A: atrium). Sparse GFP switching was also observed in the trunk (arrows). (f) Characterization of CreERT2 expression by *in situ* hybridization in *Tg(Smarcd3-F6:CreERT2)* embryos. *creert2* RNA was detected in the anterior lateral regions (c, arrow head) at 10 hpf, similar to GFP expression in *Tg(Smarcd3-F6:GFP)* embryos. Staining in heart cones was seen at 22 hpf, but could not be detected at 28 hpf. (g) Gating strategy used for sorting GFP+ cells from *Tg(Smarcd3-F6:GFP)* embryos. All scale bars shown represent 100 μm.

.


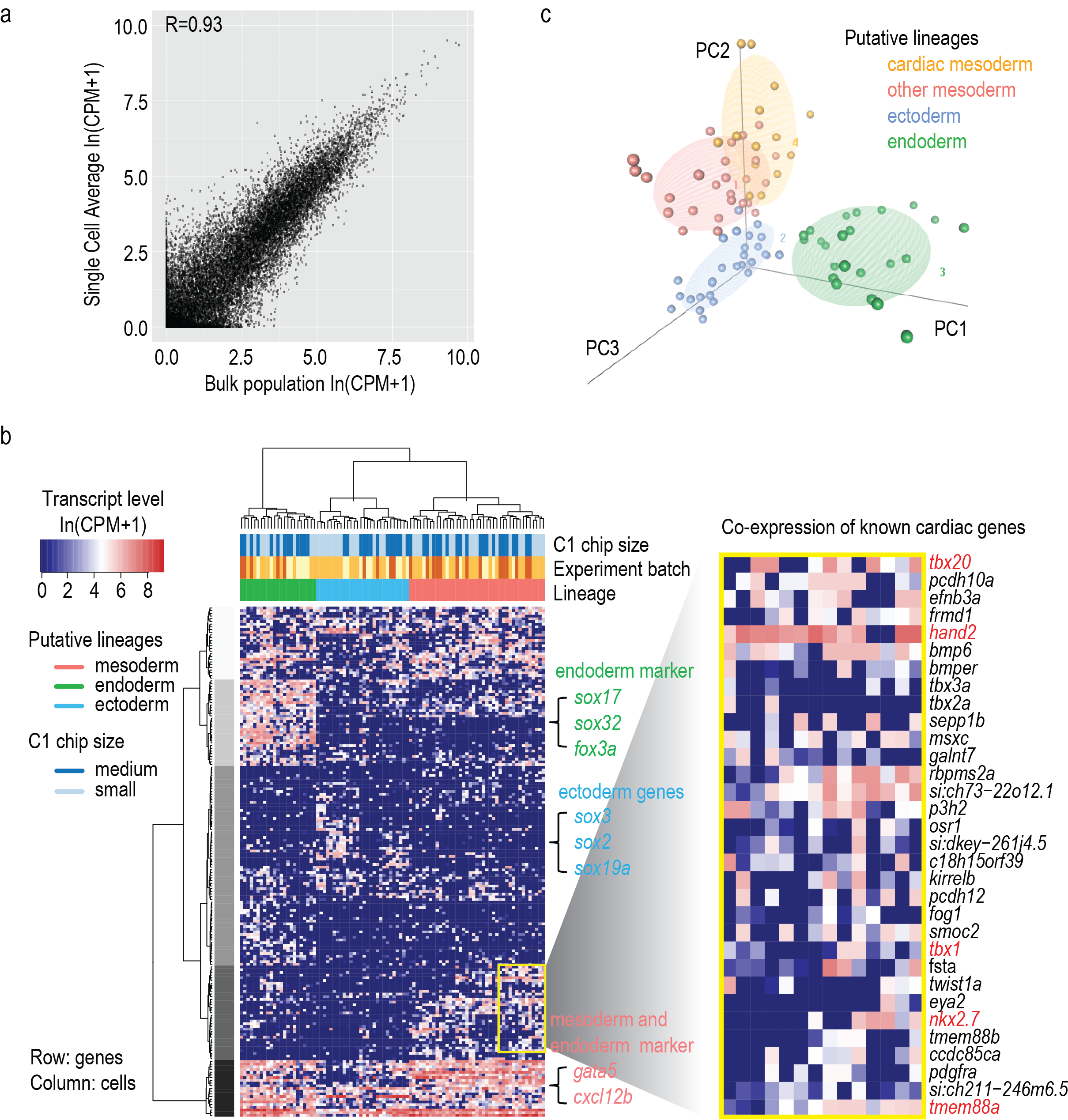


**Supplementary Figure 2**

**Single-cell mRNA-seq results of *Smarcd3*-F6:GFP+ cells.**

(a) Transcript levels of each gene in averaged single-cell transcriptome (y axis) and in *Smarcd3* enhancer labeled bulk population (x axis) were plotted. Pearson correlation coefficient of the two datasets is shown at the upper left corner**.** (b) Unsupervised hierarchical clustering with differentially expressed genes divided *Smarcd3-F6+* population into three major groups, representing putative endoderm (green), ectoderm (blue) and mesoderm (red) lineages (See methods for details). 92 cells that had at least 2000 genes detected (CPM > 0) were kept for clustering. Each row represents a gene, each column a cell. The yellow rectangle indicates the potential cardiac progenitors co-expressing known cardiac genes (gene names in red). (c) Principle component analysis with the same set of differentially expressed genes yields similar clustering results. Potential cardiac cells highlighted in the yellow rectangle in the heatmap (b) were marked as cardiac mesoderm in this plot. Note that cardiac mesoderm cells occupied a domain partially separated from the other mesoderm lineages.

**
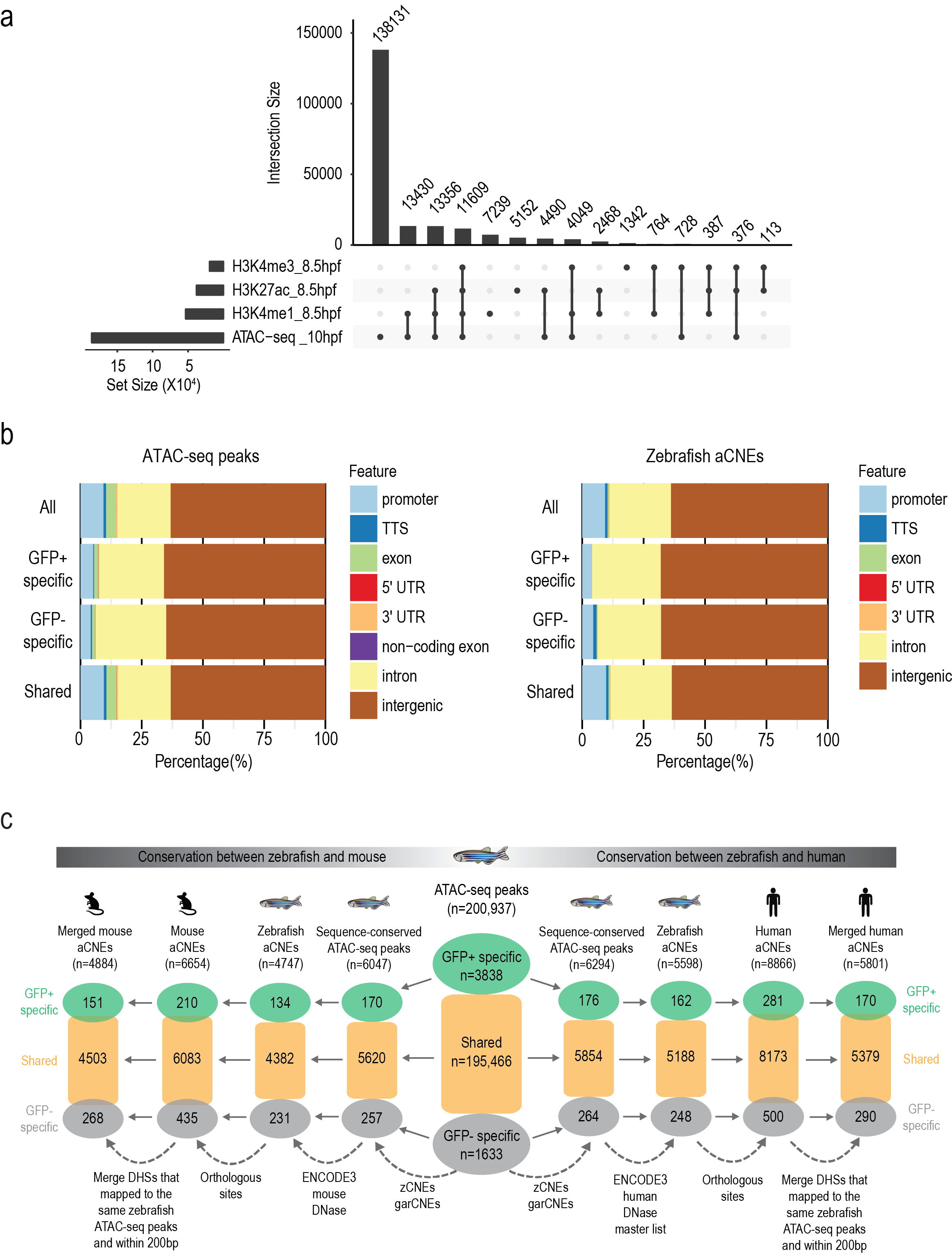
**

**
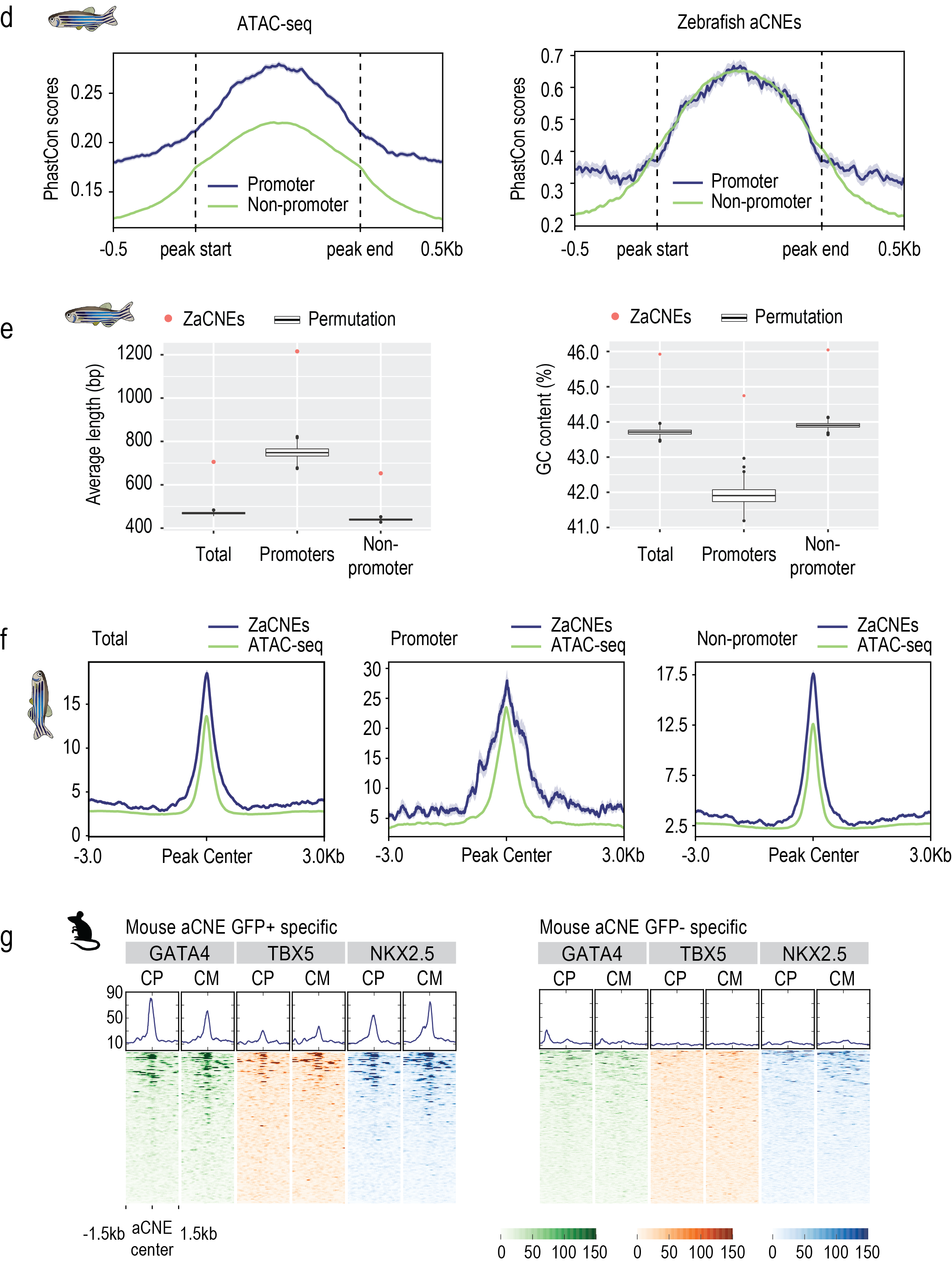
**

**Supplementary Figure 3**

**Features of ATAC-seq and aCNEs.**

(a) UpSet plot showing the comparison between previously published histone ChIP-seq data in whole zebrafish embryos of 8.5 hpf ^3^ with our ATAC-seq data. (b) Genomic distribution of ATAC-seq peaks (n= 200,937) and zebrafish aCNEs (n=4496, conserved with both human and mouse). The total dataset (all) as well as the subsets (GFP- specific, GFP+ specific, shared) was shown for both. TTS: transcription termination site (c) Workflow for identifying aCNEs and the number of aCNEs identified. GFP+ specific (green), GFP- specific (grey) and shared (orange) zebrafish aCNEs were three mutually exclusive sets of regions; however due to the non-unique mapping between human and zebrafish aCNEs, a small number of shared human aCNEs overlapped with GFP+ specific and GFP- specific human aCNEs. Merged human aCNEs were used for GREAT enrichment analysis to avoid introducing statistical bias. The same workflow was carried out for identifying aCNEs conserved between zebrafish and mouse (left panel). (d) Aggregate plot showing the phastCon scores of promoter (n=18,927) and non-promoter (n=182,010) ATAC-seq peaks, or promoter (n =420) and non-promoter (n=4076) zebrafish aCNEs, with 500 bp flanking regions included at both directions. Dotted lines indicate peak boundaries. (e) Permutation analysis comparing the average interval length and average GC content of the zebrafish aCNEs (red dot) with that of the same number of randomly selected ATAC-seq peaks (box plot, distribution of the results from 1000 times permutation). Results for total, promoter and non-promoter aCNEs are shown. Same scheme of boxplot was used as in Fig. 5 (f) Aggregate plot of ATAC-seq signals centered on zebrafish aCNE midpoints (blue) or ATAC-seq peak midpoints (green). Results for total, promoter and non-promoter aCNEs/ATAC-seq peaks comparison are shown. The Wilcoxon test (two-sided) was used to determine if the open chromatin signals between aCNEs and ATAC-seq peaks were different (Total: *P* = 2.84e-271; promoter: *P* = 1.31e-10; non-promoter: *P*=2.80e-276) (g) Heatmap showing the binding of cardiac TFs to mouse aCNEs conserved with GFP+ or GFP- specific ATAC-seq peaks. Read intensities from cardiac TF ChIP-exo data^1^ were plotted for 3kb regions centered on aCNE midpoints. CP: cardiac precursors, CM: cardiomyocytes.


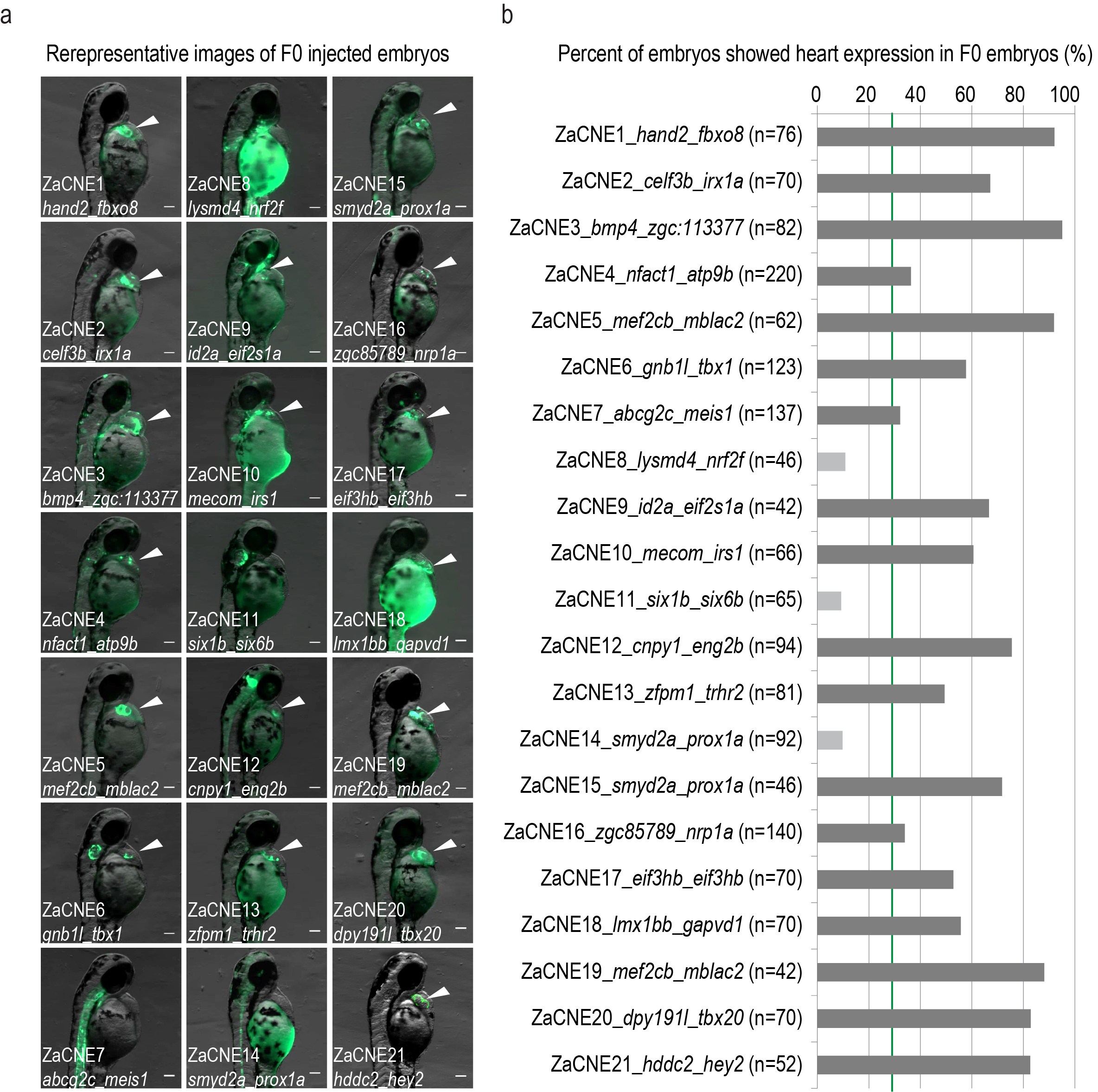


**Supplementary Figure 4**

**Results of enhancer reporter assay in F0 generation.**

Representative images (a) and scoring of heart expression (b) of F0 embryos injected with ZaCNE GFP reporter constructs. The two closest genes near each ZaCNEs (assigned by GREAT V 3.0.0) were indicated. Green line in (b) indicates the 30% threshold. Enhancers that did not pass the threshold were colored in light grey (ZaCNE8, ZaCNE11 and ZaCNE14). All images were taken on transgenic embryos of 48-52 hpf. All scale bars represent 100 μm.

**
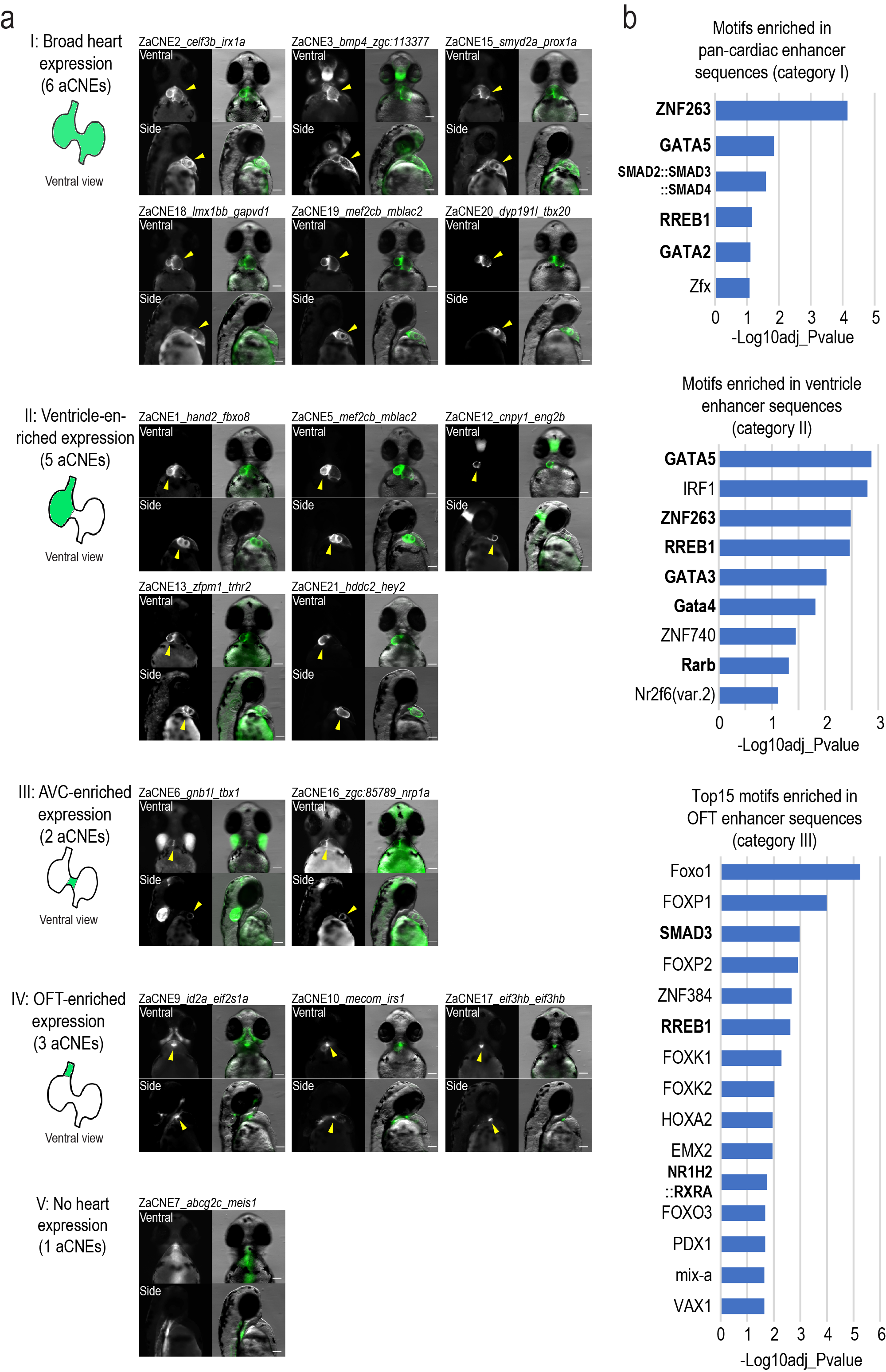
**

**
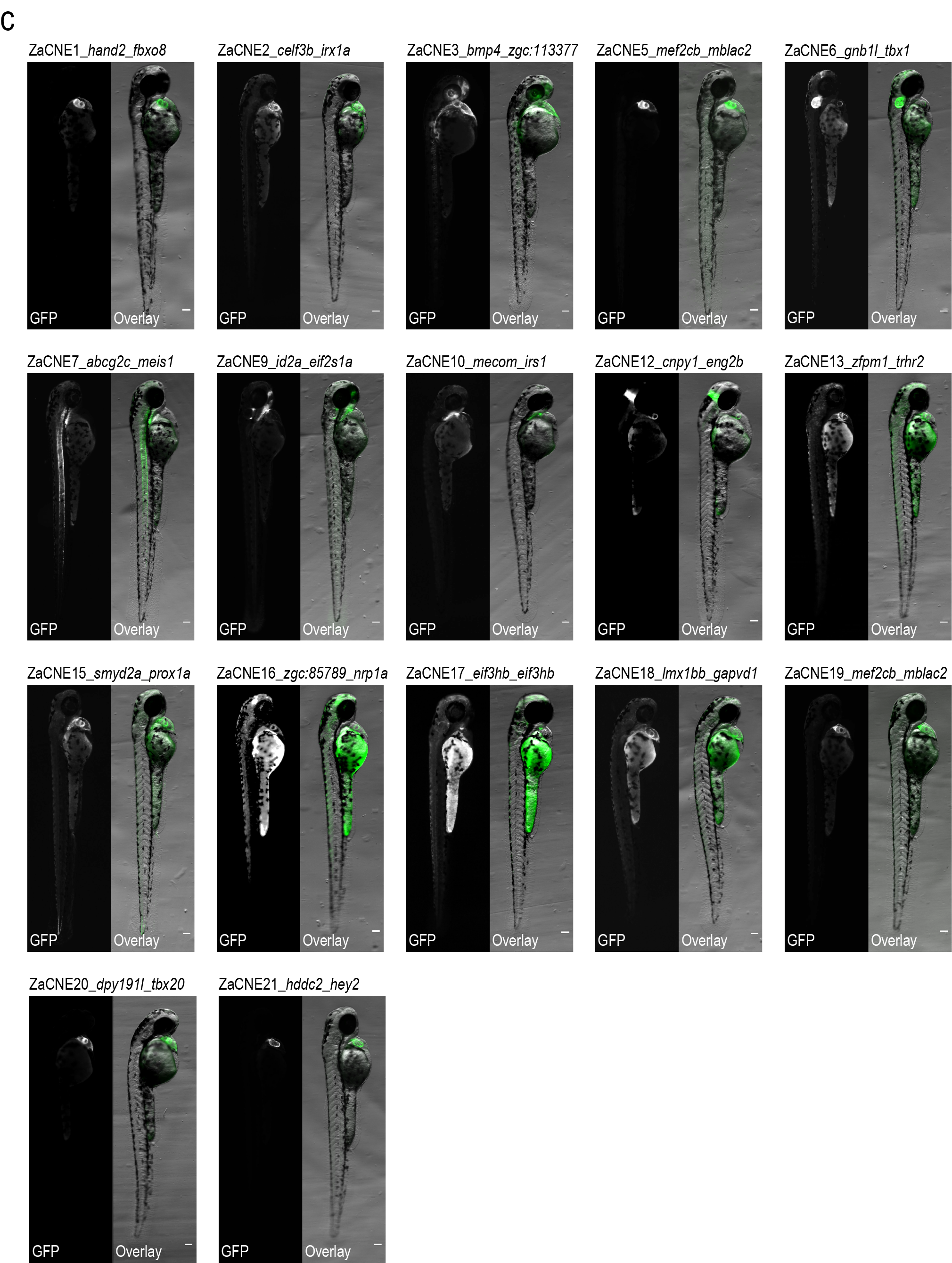
**

**
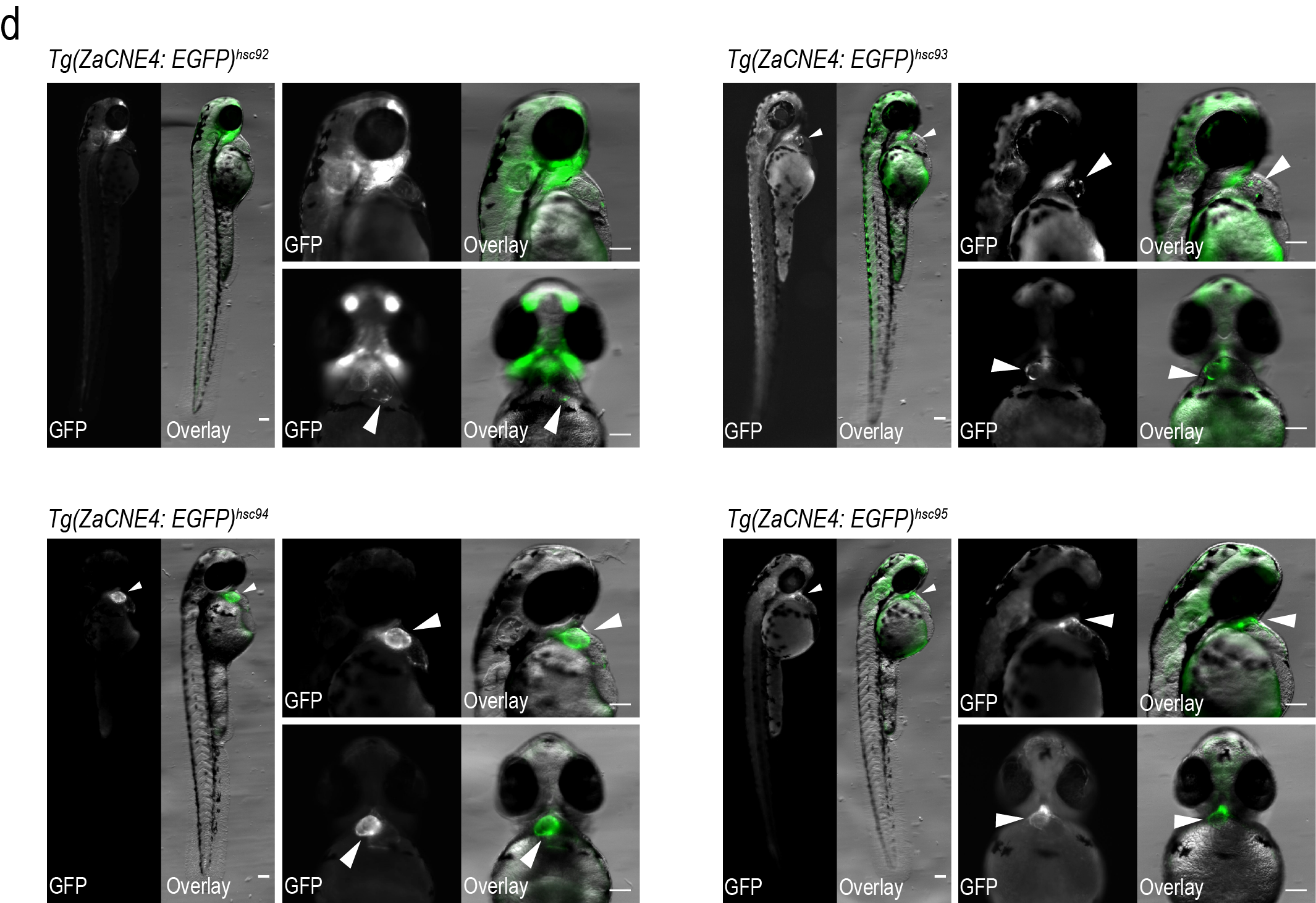
**

**
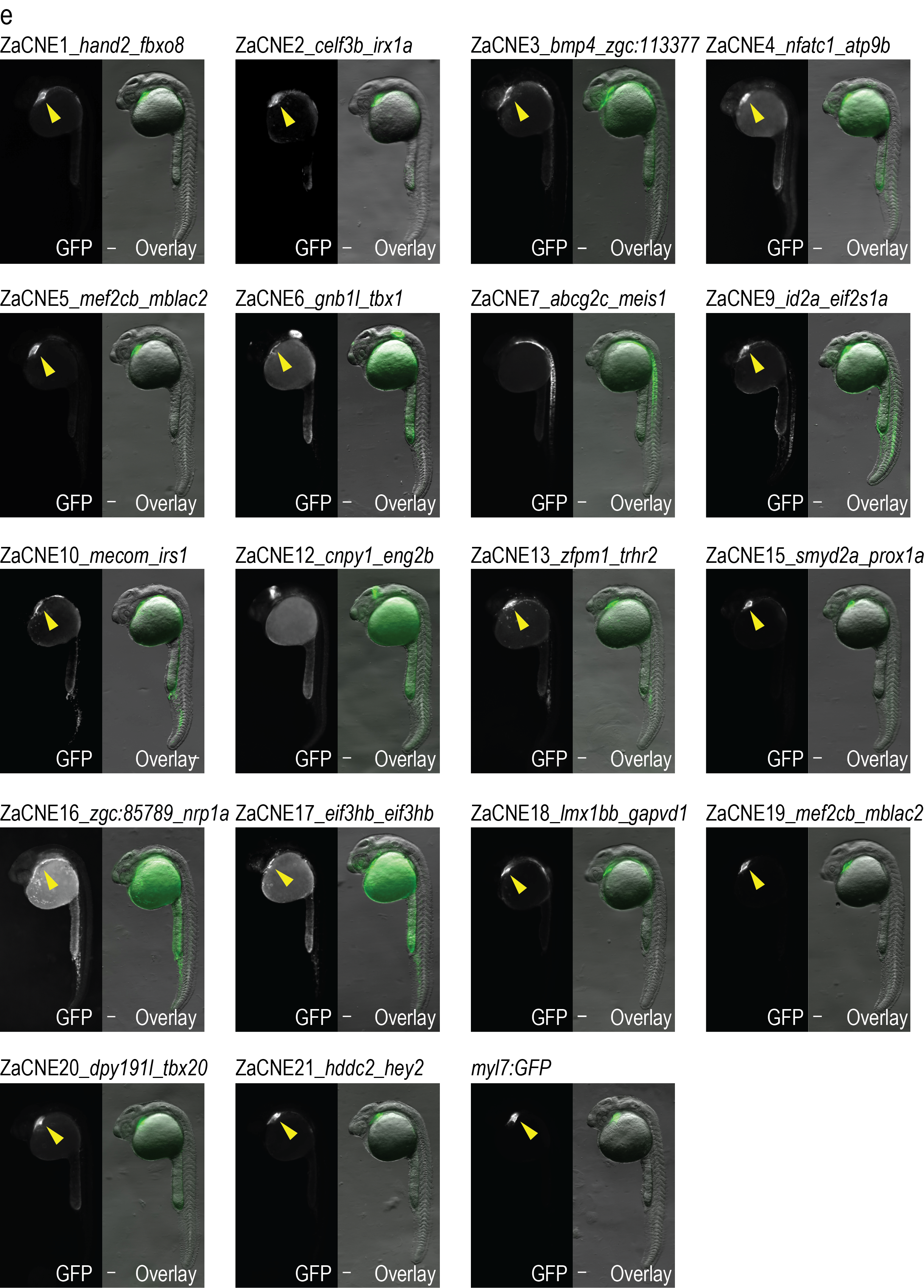
**

**Supplementary Figure 5**

**Results of enhancer reporter assay in F1 generation.**

(a) Cardiac expression patterns observed in 17 ZaCNEs enhancer stable lines were grouped into 4 categories (as shown in Fig. 3). For each enhancer, representative images of both ventral (up) and side (down) views were shown in GFP only (left) and GFP-brightfiled overlay channels (right). Expression patterns driven by ZaCNE4 lines were shown separately in panel (d). (b) Motifs enriched in enhancers of category I (pan-cardiac), II (ventricle-enriched) and III (OFT-enriched). All enriched motifs in category I and II and top15 enriched motifs in category III were shown. Bold letter indicated motif enrichment shared by different categories. (c) Whole-body view of the *Tg(ZaCNE: EGFP)* stable lines (except ZaCNE4 which was shown in panel d). (d) Multiple different expression patterns were observed in *Tg(ZaCNE4:EGFP)* transgenic lines. 4 alleles have been identified for this ZaCNE. Though showing 4 distinct expression patterns, all 4 alleles drive heart expression to a certain degree. It is likely that ZaCNE4 is a weak heart enhancer whose activity tend to be affected by the regulatory elements near the transgene integration loci. All images in (a) (c) (d) were taken on transgenic embryos of 48-52 hpf. (e) Images of 18 stable ZaCNE transgenic lines at 22-24 hpf. An image of *Tg (myl7:GFP)^twu34^* line was used as a positive example to show the linear heart tube at 24 hpf. Yellow arrowheads indicate expression of GFP in linear heart tubes. All scale bars represent 100 um.

**
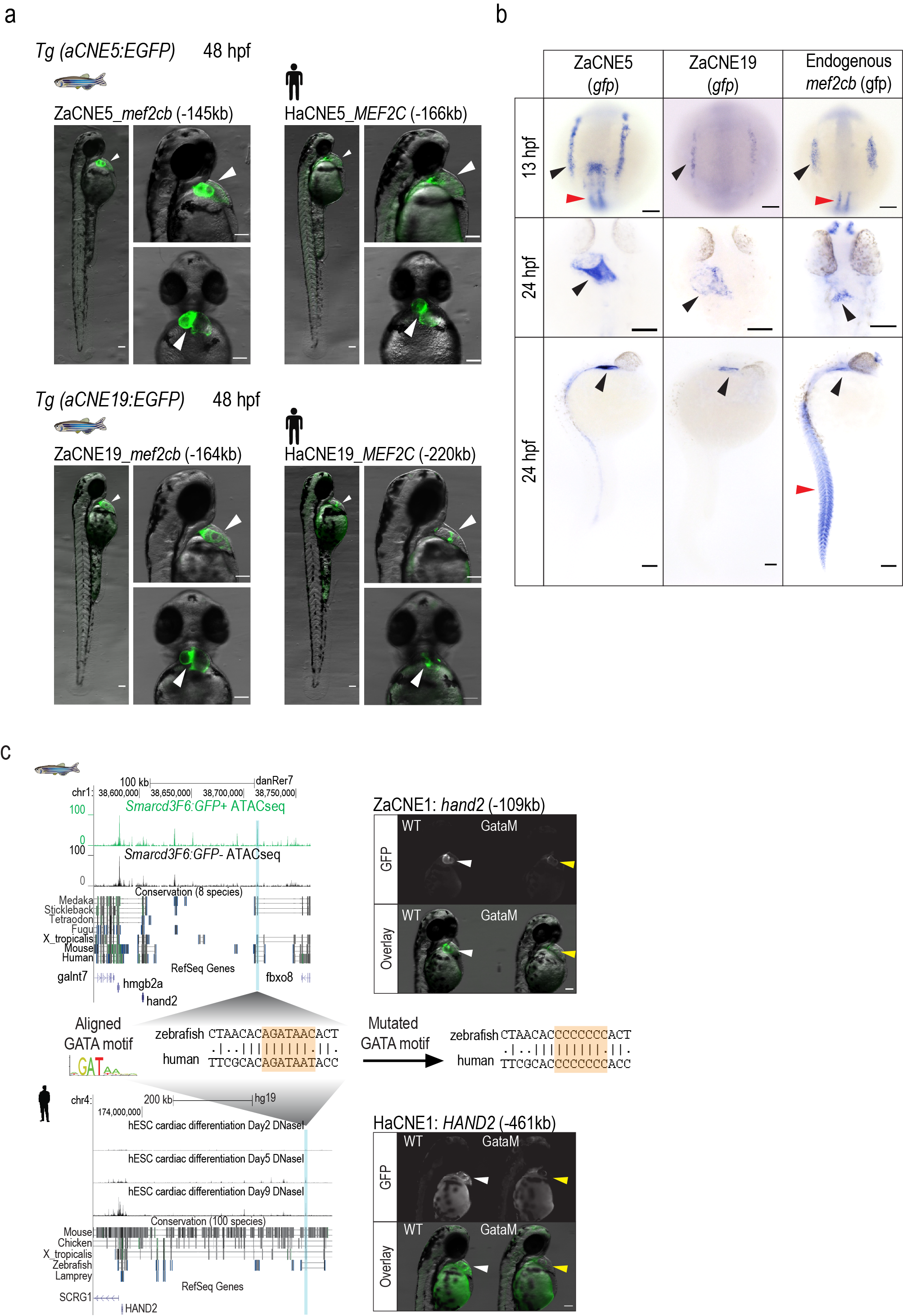
**

**Supplementary Figure 6**

**Zebrafish and human orthologous aCNEs share conserved cardiac activities.**

(a) Fluorescent images of stable GFP transgenic lines generated using zebrafish or human aCNE sequences. (b) *In-situ* characterization of ZaCNE5 and ZaCNE19 enhancer activity and endogenous *mef2cb* expression. Black arrowheads indicate cardiac expression and red arrowheads show expression in somites. (c) Cardiac activity of the aCNE1 depends on the binding of GATA factors. (Left) genome browser view showing the location of the aCNE1 enhancers (cyan shaded) in human and zebrafish genome. Both aCNE1 enhancers were located a few hundred kilobases upstream of the *hand2/HAND2* gene. An aligned GATA motif exists in the aCNE1 enhancers. (Right) mutation of the GATA motif within aCNE1 enhancers greatly decreased their cardiac activities. Images were taken on F1 embryos of the transgenic lines generated using the WT or GATA motif mutated enhancer sequences. Images in (a) and (c) were taken on embryos of 48-52 hpf.


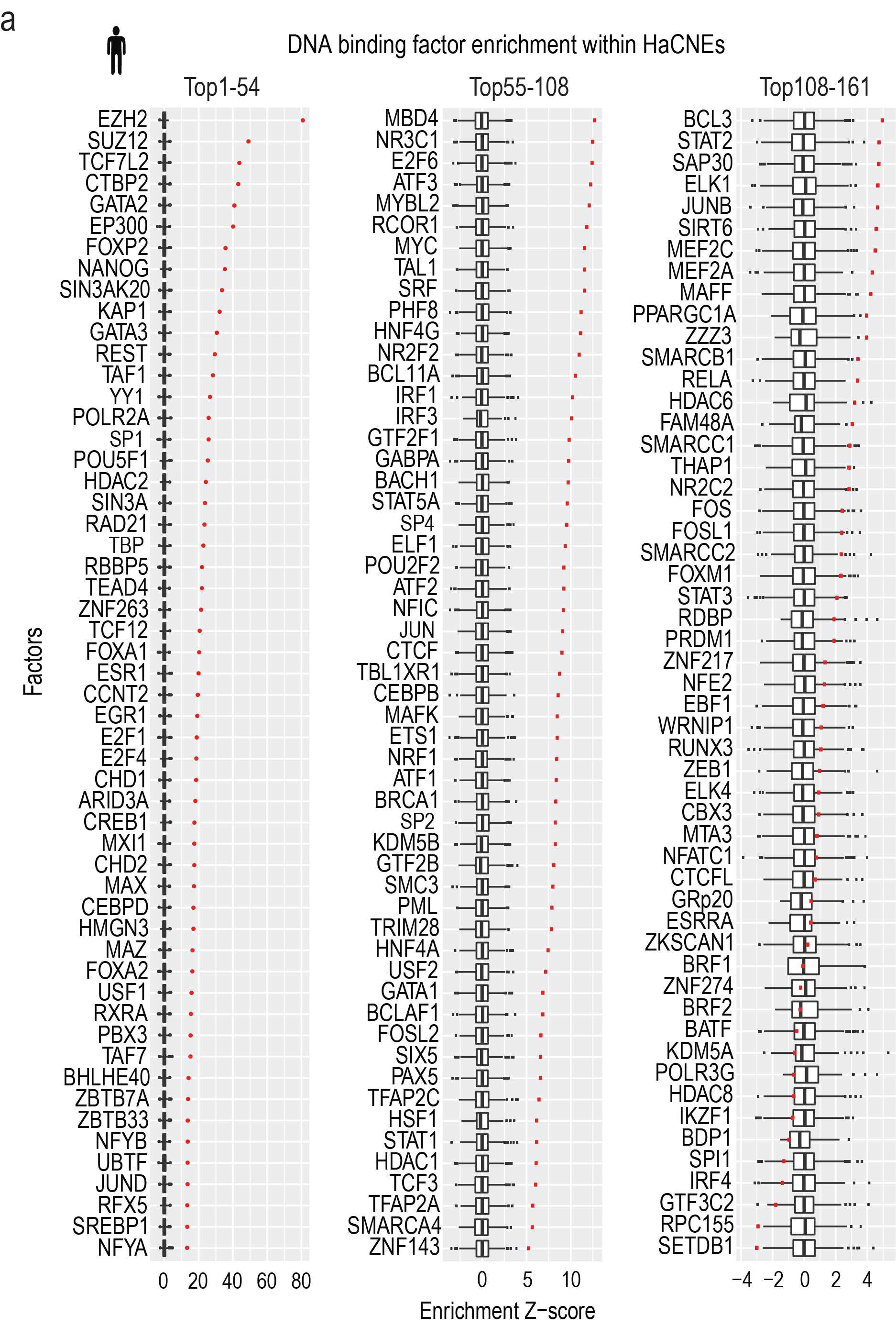


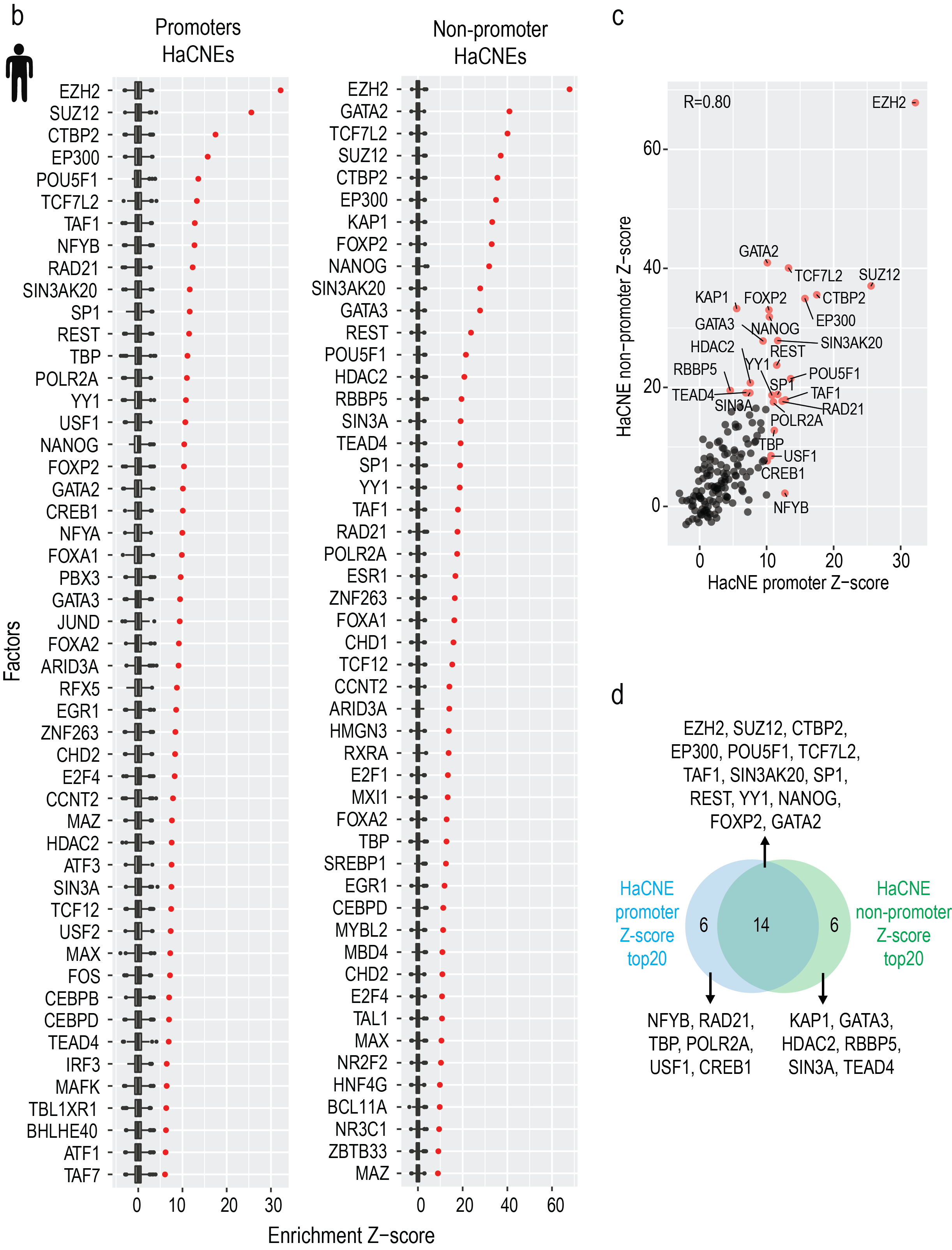


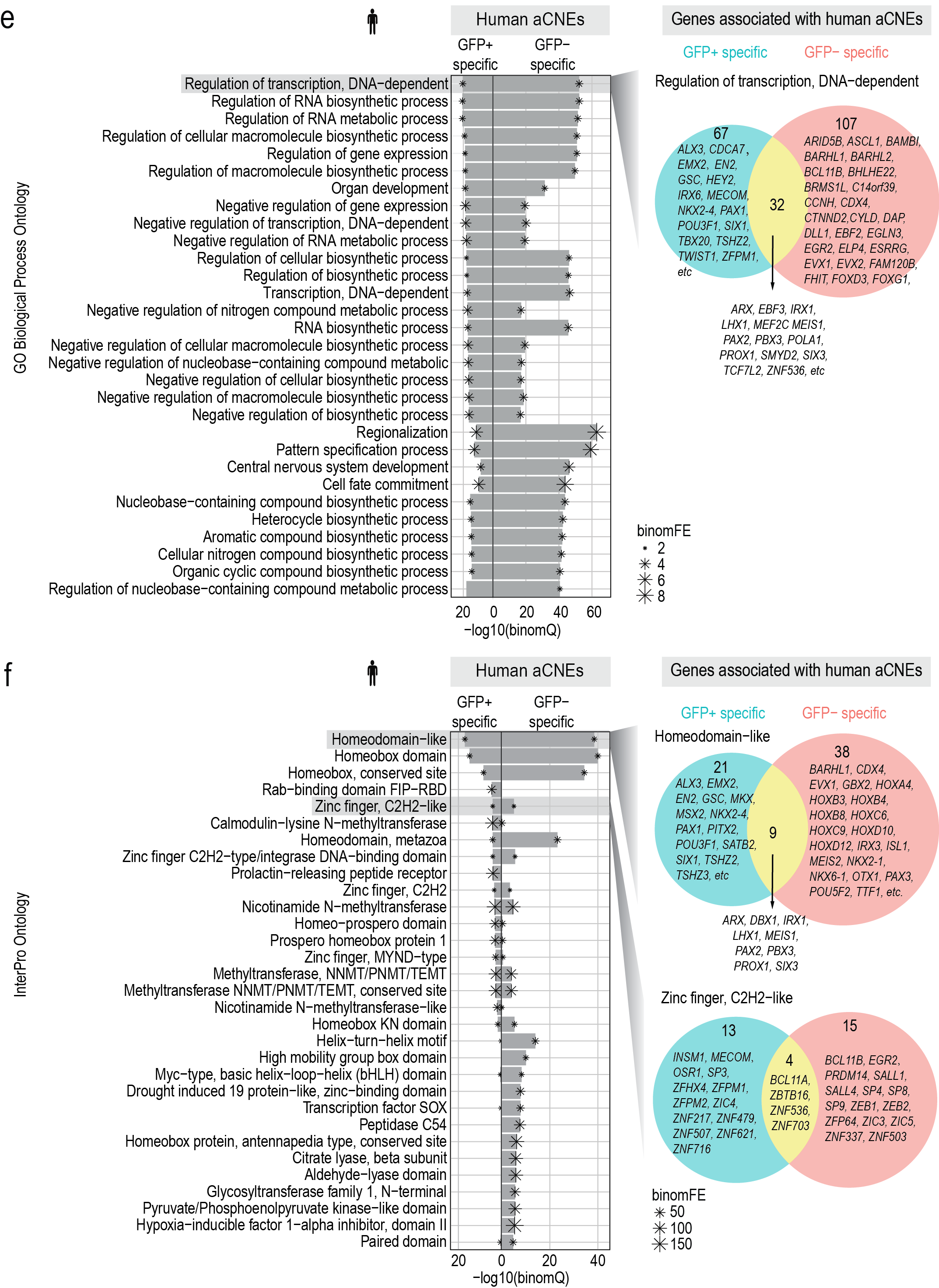


**Supplementary Figure 7**

**DNA binding factor and functional enrichment within the 8866 human aCNEs.**

(a) Full enrichment results of the 161 DNA binding factors in human aCNEs (see Methods for details), as shown in Fig. 5b. (b) Top 50 enriched DNA binding factors in promoter human aCNEs (n=1089) and non-promoter human aCNEs (n=7777). (c) Scatter plot showing the enrichment Z-score in promoter aCNEs and non-promoter aCNEs for the 161 factors. Pearson correlation coefficient between the two sets of Z-scores was shown at the left corner. Factors with top 20 enrichment Z-scores in promoter or non-promoter aCNEs were colored in red. (d) Venn diagrams showing the overlap of the top 20 enriched DNA binding factors in promoter aCNEs and non-promoter aCNEs. (e) (f) GREAT Enrichment analysis of human aCNEs conserved with GFP+ or GFP- specific ATAC-seq peaks, as shown in Fig. 5A. Top 20 enriched terms (binomial FDR < 0.5, FE >2, sorted by binomial FDR) from ‘GO Biological Process’ (e) and ‘InterPro’ (f) for each category (GFP+, GFP-) were plotted. Venn diagrams showing the overlaps of genes associated with the GFP+ and GFP- human aCNEs that contribute to the enrichment of the ontologies. In all boxplots, center represents median, lower and higher hinges correspond to the first and third quartiles, and whiskers extend to values no further than 1.5 *(distance between first and third quantile) from the hinges. Data beyond the end of whiskers are plotted individually as outliers. FE: fold of enrichment; FDR: false discovery rate

**
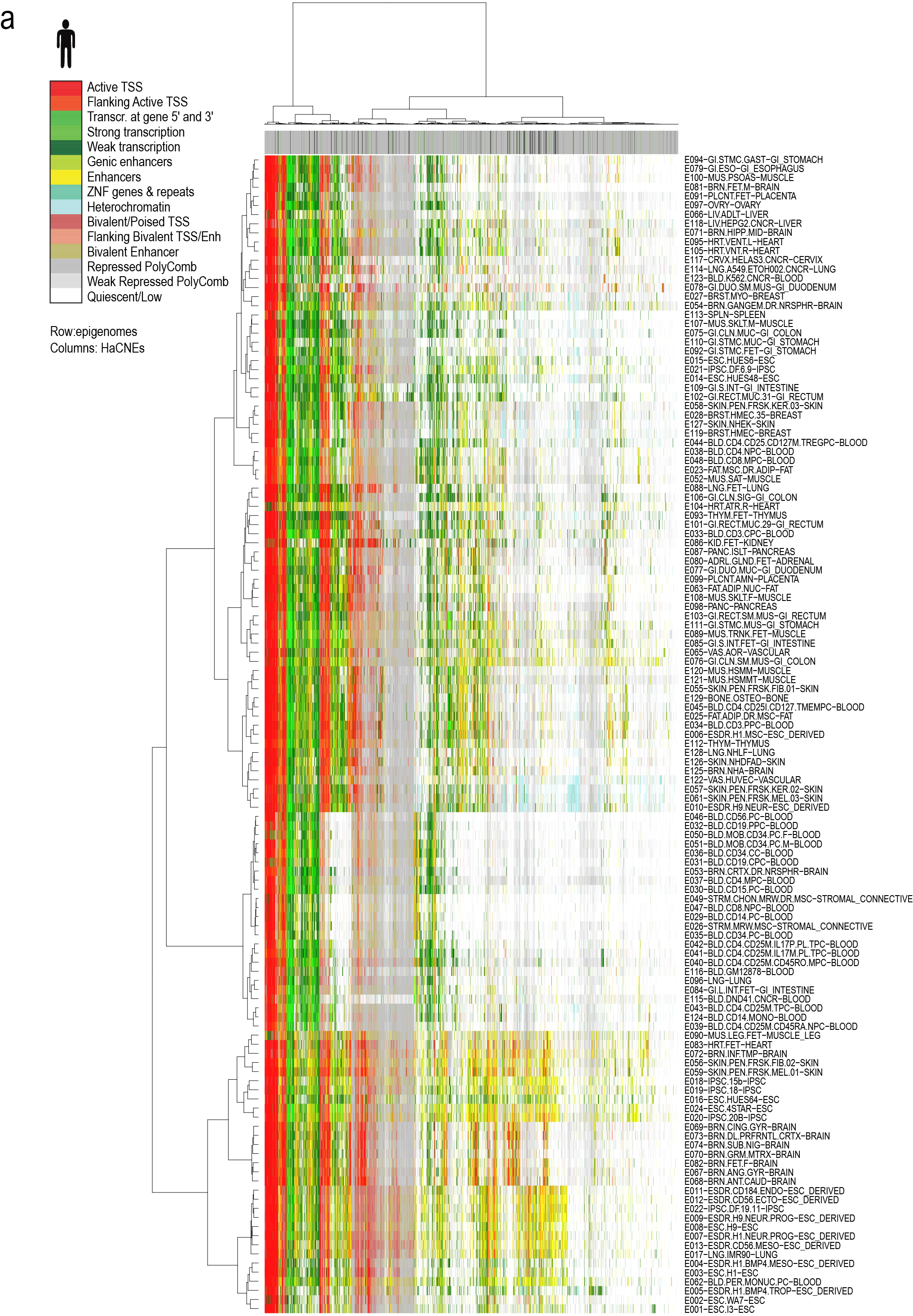
**

**
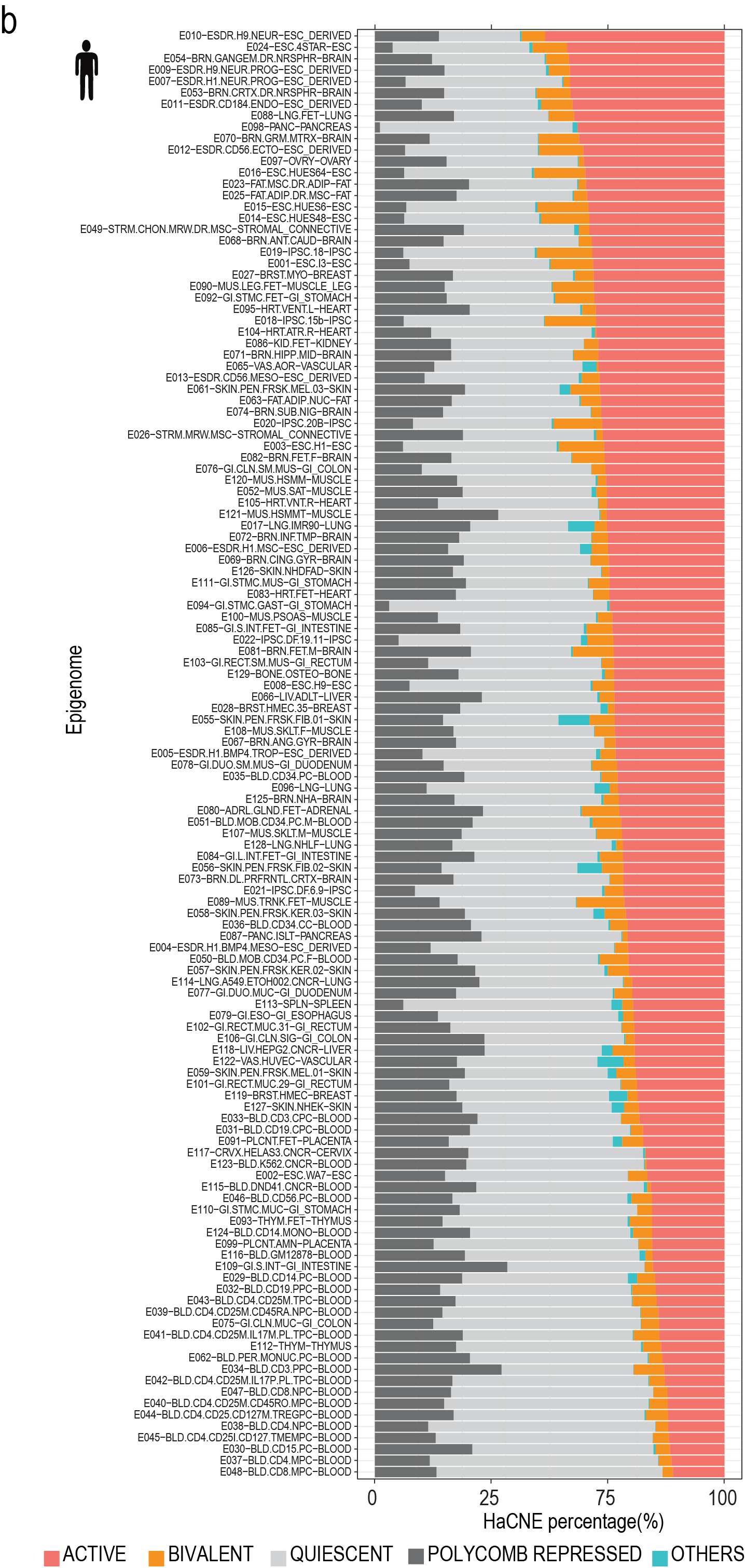
**

**
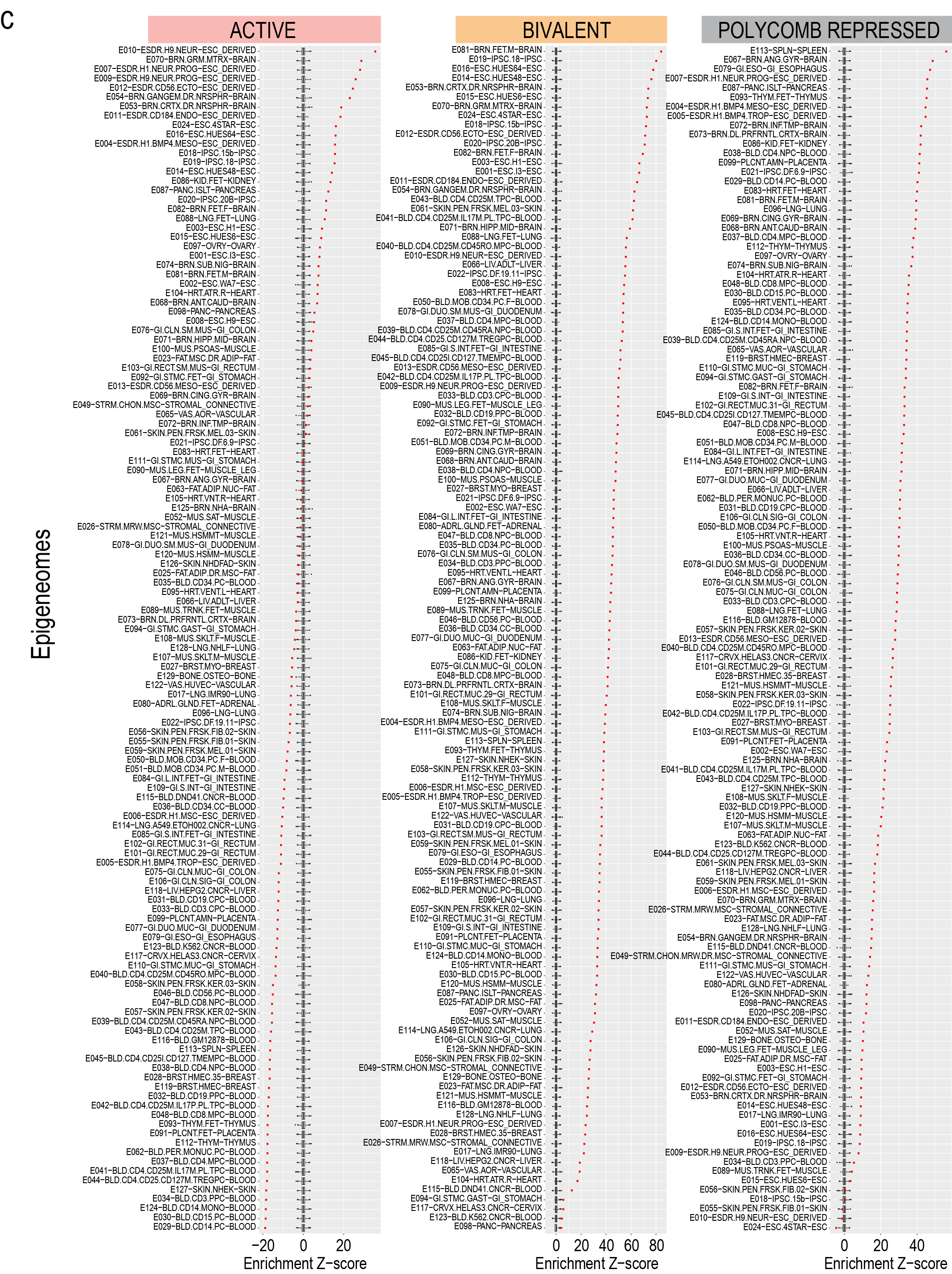
**

**
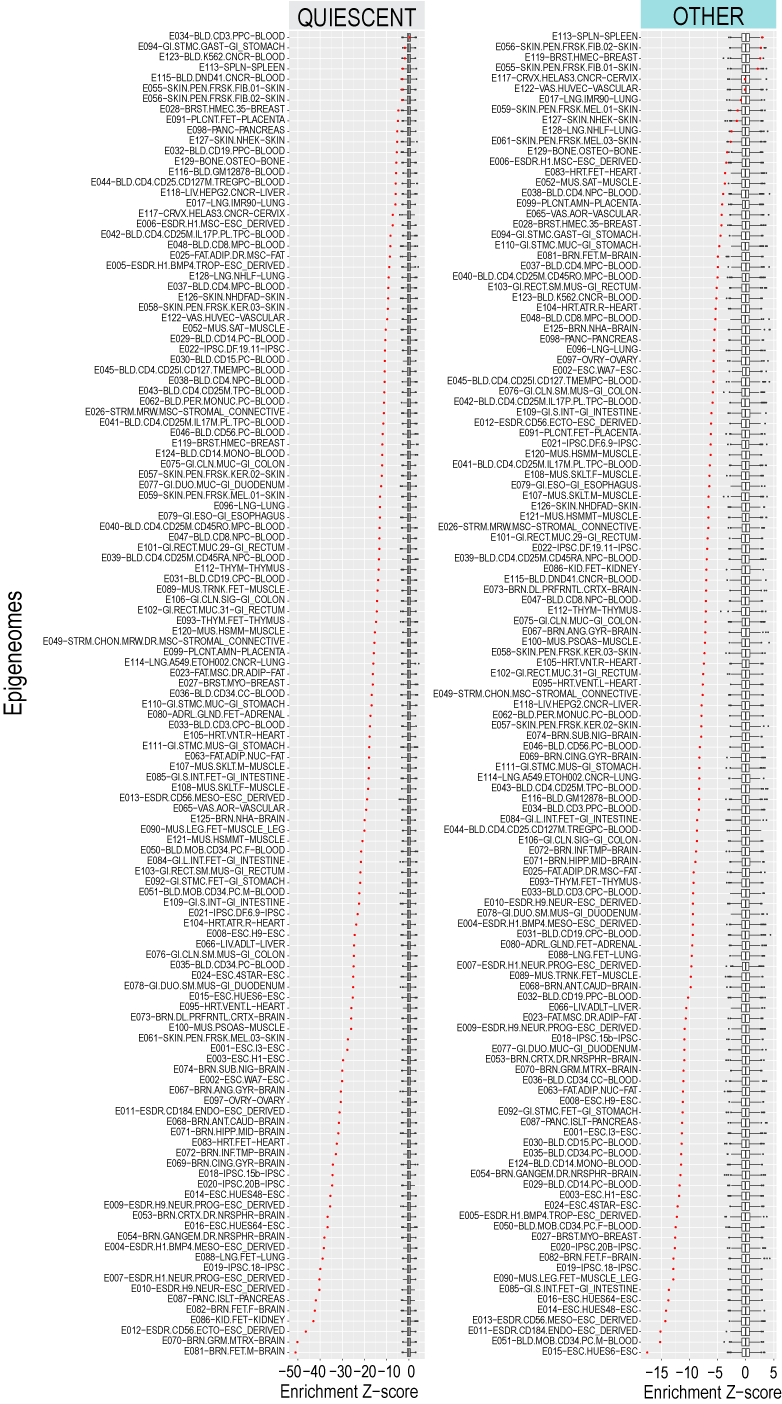
**

**Supplementary Figure 8**

**Chromatin states of human aCNEs in 127 epigenomes**

(a) Heatmap showing the chromatin state of 8866 Human aCNEs in 127 epigenomes retrieved from the Roadmap Epigenomics Project^4^, as shown in Fig. 5C. (b) Percentage of Human aCNEs (n=8865) showed Active, Bivalent, Polycomb repressed, Quiescent and Other states in the 127 epigenomes. Epigenomes were sorted based on the number of Active HaCNEs they possessed. Note that ESC-derived neuronal progenitors, ESCs and brain tissues have the highest numbers of Active HaCNEs while hematopoietic lineages have least. (c) Full enrichment results of aCNEs for regions of different chromatin states (Active, Bivalent, Polycomb repressed, Quiescent and Other) in all 127 epigenomes, as shown in Fig. 5f. In all boxplots, center represents median, lower and higher hinges correspond to the first and third quartiles, and whiskers extend to values no further than 1.5 *(distance between first and third quantile) from the hinges. Data beyond the end of whiskers are plotted individually as outliers.

**Supplementary Table 1**

**Breakdown of CNEs used in our analysis**

|  | zCNE (Hiller et al, 2013) | | Extra ones added from Gar CNE (Braasch et al, 2016) | Total zebrafish CNEs |
| --- | --- | --- | --- | --- |
|  | direct | Indirect | indirect | Both direct and indirect |
| CNE with mouse | 9429 | 2145 | 7253 | 18827 |
| CNE with human | 10187 | 2109 | 7709 | 20005 |

**Supplementary Table 2:**

**Numbers of all ATAC-seq peaks, sequence-conserved ATAC-seq peaks and aCNEs**

|  | All | Sequence-conserved | | | Zebrafish aCNEs | | | |
| --- | --- | --- | --- | --- | --- | --- | --- | --- |
|  |  | with mouse | with human | with both | with mouse | with human | with both | With human or mouse |
| All | 200937 | 6047 | 6294 | 5934 | 4747 | 5598 | 4496 | 5849 |
| GFP+ specific | 3838 | 170 | 176 | 166 | 134 | 162 | 125 | 171 |
| GFP- specific | 1633 | 257 | 264 | 254 | 231 | 248 | 221 | 258 |
| Shared | 195466 | 5620 | 5854 | 5514 | 4382 | 5188 | 4150 | 5420 |

**Supplementary Table 3**

**Numbers of mouse and human DHSs (all, sequence-conserved, aCNEs)**

|  | All DNase I sites | Sequence-conserved | aCNEs |
| --- | --- | --- | --- |
| Mouse | 1199722 | 12326 | 6654 |
| Human | 4193929 | 17477 | 8866 |

**Supplementary Methods**

**Zebrafish injection and transgenic lines**

2.7 kb Mouse *Smarcd3*-F6 sequence (mm9 assembly chr5: 24113559 -24116342)^5^ was sub-cloned from a gateway entry vector into Zebrafish Enhancer Detection (ZED) Vector^6^ and *E1b-Tol2-GFP-gw* vector^7^ (Addgene #37846) using the Gateway recombination system (Invitrogen, Gateway LR Clonase II Enzyme Mix, Cat# 11791020). Tol2 mRNA was synthesized using mMessage mMachine kit (Ambion, Cat# 12538120) and purified by MEGAclear™ Transcription Clean-Up Kit (Ambion, Cat# AM1908). 25ng ZED or *E1b-Tol2-GFP-gw* plasmid carrying the *Smarcd3*-F6 enhancer was microinjected into wildtype embryos at the one-cell stage with 150 ng Tol2 mRNA following the standard Tol2 procedure^8^. 2 independent germline carriers have been identified for *Tg (Smarcd3-F6: EGFP)* generated using *ZED-Smarcd3-F6* plasmid and 4 independent carriers have been identified from F0 founders generated using *E1b-Tol2-GFP-Smarcd3-F6* plasmid. Transgenic embryos from different carriers display similar GFP expression in heart. 4 alleles (*Smarcd3-F6:EGFP^hsc70^, Smarcd3-F6:EGFP^hsc71^, Smarcd3-F6:EGFP^hsc72^, Smarcd3-F6:EGFP^hsc73^*) have been raised (see Supplementary Data 4 for details). A line (*Smarcd3-F6:EGFP^hsc70^*) with brighter GFP expression (generated using ZED-Smarcd3-F6 plasmid) was used for all related figures presented. Since we noticed maternal expression of GFP in the *Tg (Smarcd3-F6: EGFP)* line, we only used male *Tg (Smarcd3-F6: EGFP)* fish crossed to female wildtype fish for our experiments.

To generate a *Tg(Smarcd3-F6:CreERT2)* line, we adopted two strategies. In the first one, EGFP sequence in *E1b-Tol2-GFP-gw* vector was first replaced by CreERT2 sequence to generate an *E1b-Tol2-CreERT2-gw* vector. The *Smarcd3*-F6 enhancer was sub-cloned into this CreERT2 vector via Gateway recombination. In the second strategy, the *Smarcd3*-F6 enhancer was sub-cloned into a gateway 5’ entry vector (*p5E-Smarcd3-F6*). A middle entry vector containing the *gata2* minimal promoter that was used in the ZED vector (*pME-gata2P*) and a 3’ entry vector with CreERT2 (*p3E-CreERT2*) were also generated. *p5E-Smarcd3-F6, pME-gata2P* and *p3E-CreERT2* were recombined with *pDestTol2-cCrystalGFP* to generate *pDestTol2cCrystalGFP:Smarcd3-F6-gata2P-CreETR2* construct (Invitrogen, LR Clonase II Plus Enzyme, Cat# 12538120). All gateway entry vectors used were from the Tol2 kit^9^. Microinjection was carried out in the same way as for *Tg (Smarcd3-F6: EGFP)* line. 5 independent lines from *E1bTol2CreERT2:Smarcd3-F6* and 2 independent carriers from *pDestTol2cCrystalGFP:Smarcd3-F6-gata2P-CreETR2* were identified. Eventually 5 different alleles (*Smarcd3-F6:CreERT2^hsc74^, Smarcd3-F6:CreERT2^hsc75^, Smarcd3-F6:CreERT2^hsc76^, Smarcd3-F6:CreERT2^hsc81^, Smarcd3-F6:CreERT2^hsc88^*) were raised (see Supplementary Data 4 for details). Lineage tracing results seen in different *Tg(Smarcd3-F6:CreERT2)* lines were not obviously different. Results presented in Supplementary Fig. 1 were generated from embryos of *Tg (Smarcd3-F6:CreERT2)^hsc76^*. The *Tg(nkx2.5: ZsYellow) ^fb7^*, *Tg(βactin2:loxP-DsRed-STOP-loxP-EGFP)^s928Tg^* and *Tg (myl7:GFP)^twu34^* lines used were generated in previous studies^10–12^.

**Processing and analysis of mRNA-seq data**

Raw reads were analyzed with FastQC (version 0.11.2)^13^, trimmed with Trimmomatic (version 0.32)^14^ (ILLUMINACLIP:TruSeq3-PE-2.fa:2:30:10:5:true LEADING:20 TRAILING:20 SLIDINGWINDOW:5:25 MINLEN:36) before aligned to the Zv9 zebrafish genome assembly using STAR (version 2.4.1)^15^ with default settings. The percent of uniquely mapped reads is (80±2.6)% for bulk samples and (63±9.3)% for single-cell libraries (see Supplementary Data 6 for details). HTSeq-count (version 0.6.0)^16^ and Zv9 (Ensembl release 79) transcriptome annotation were used to determine the number of reads mapped to each gene. An average of 14486 genes were detected in bulk samples and 3901 genes in single cells.

For bulk mRNA-seq, genes that have at least 1 read per million in at least 2 replicates were kept for downstream analysis. edgeR package (version 3.18.1) was used for normalization and differential gene expression analysis^17^. 167 and 147 genes were identified as more highly expressed in GFP+ and GFP- cells respectively (FDR < 0.05, Fold change > 2). Volcano plot showing differentially expressed genes was generated with an in-house R script.

Functional enrichment analysis was performed using online tool g:Profiler^18^. A more stringent list of differentially expressed genes (FDR < 0.05, Fold change > 4) were ordered based on fold change and then used as the input for g:Profiler. Only functional categories containing more than 2 genes, but less than 500 genes were included in our analysis. Benjamini-Hochberg FDR method was used for multiple testing correction to adjust significance thresholds. The top 10 enriched GO terms (sorted by FDR) for each gene list were plotted.

For single-cell data, 92 cells with more than 2000 genes detected (counts per million, CPM >0) were kept for clustering analysis. Gene counts (CPM) for each cell were first normalized using TMM method implemented in edgeR package^17^ and then winsorized before log transformation (In(CPM+1)). To perform unsupervised clustering, 189 genes were selected and used, if they were differentially expressed in bulk mRNA-seq (|log2FC|>1.5, FDR < 0.1) and have an expression level (In(CPM+1) > 4) in at least one single cells. The Manhattan distance and ward.D method were used for unsupervised clustering.

**ATAC-seq reads mapping and differential peak identification**

Raw reads were preprocessed by FastQC (version 0.11.2)^13^ and Trimmomatic (version 0.32)^14^ (LEADING:20 TRAILING:20 SLIDINGWINDOW:5:25 MINLEN:36) before being aligned to Zv9 zebrafish genome assembly by BWA (version 0.7.8) under aln model^19^. Reads with mapping quality score > 30 were kept for downstream analysis using SAMtools (version 1.2) (samtools view -b -q 30). Two replicates for each sample were merged for peak calling by MACS2 (version 2.7.9)^20^ (--nomodel --nolambda --gsize 1.4e9). Around 150,000 peaks were identified in both GFP+ and GFP- populations. Peaks showing enriched signals in one population versus the other (GFP+/- specific peaks) were identified using DiffBind package (version 2.6.6)^21^ (DEseq2, FDR < 0.05). DiffBind first generated a consensus peak set that represent an overall set of candidate open chromatin regions based on all four samples (both GFP+ and GFP- samples). Then it counted if the sequence mapped to each interval in the consensus peak set were significantly different in GFP+ and GFP- samples. After DiffBind analysis, we obtained a total of 200,937 ATAC-seq peaks (consensus peak set), within which 3838 were GFP+ specific peaks and 1633 were GFP- specific. We used the output from DiffBind for downstream analysis.

**ATAC-seq peak annotation**

The genomic distribution annotation of ATAC-seq peaks was conducted using the Homer (version 4.9)^22^ annotatePeaks function and based on danRer7 RefSeq database. Default settings from Homer were used except that promoter was defined as 3 kb upstream to 1 kb downstream of a transcription start site (TSS). To compare the genomic distribution of different sets of peaks, the categories ‘exon’, ‘5' UTR exon’, ‘3' UTR exon’, and ‘non-coding exon’ were collapsed into one category that we designated ‘all exon’. The ‘all exon’ category, together with 4 additional categories ‘promoter’, ‘TTS’, ‘intron’, ‘intergenic’, were compared.  Two-sided Fisher's exact test was used to test enrichment or depletion of each category and the Bonferroni method was used for multiple test correction.

To compare the overlap of ATAC-seq peaks with previously published histone ChIP-seq data of similar stage (8.5 hpf)^3^, H3K4me3 (n=20,459), H3K27ac (n=39,744) and H3K4me1 (n=59,091) peaks determined by the authors were merged with all our ATAC-seq peaks (n=200,937) to generated a consensus ATAC-histone peak set (n=203,634). Then this consensus peak set was intersected (IntersectBed from BEDtools^23^, version 2.19.1) with the original ATAC-seq and histone peaks to obtain the consensus coordinates for each of the 4 datasets. The consensus coordinates were used for comparing the overlap between each dataset and making the UpSet plot (UpSetR^24^ version 1.3.3). The overlap significance between ATAC-seq and histone peaks was determined using GAT tool (1000 simulation)^25^ with whole genome as background.

**Enrichment analyses of ATAC-seq**

CentriMO (version 4.11.2)^26^ was used to identify motifs enriched at the center of ATAC-seq peaks. In order to obtain the summits of GFP+/- specific peaks, we first performed BEDtool-intersect between the peak summits determined by MACS2 (version 2.7.9)^20^ and the GFP+/- specific peaks identified by diffBind^21^. By doing this, we identified 3861 GFP+ specific summits and 1635 GFP- specific ones. 250 bp was extended to each side from the peak summits and the new regions with 501 bp uniform length were used as input for CentriMO. To identify motifs enriched in GFP+ or GFP- specific peaks, the same numbers of shared peaks were subsampled and used as the comparative dataset in the absolute and differential model. All other parameters were kept as default. Fisher E-values of the top 4 enriched motifs were plotted in the motif probability graphs.

GREAT (version 3.0.0) online tool^27^ was used for functional enrichment analysis of open chromatin regions. Whole genome was used as background and the basal plus extension rule was used for associating genomic regions with genes. In GREAT, each gene is assigned a basal regulatory domain of 5 kb upstream and 1k b downstream of the TSS (regardless of other nearby genes). The gene regulatory domain is extended in both directions to the nearest gene's basal domain but no more than 1 Mb in one direction. Functional terms showing a binomial FDR < 0.05 and a minimal region-based fold of enrichment of 2 were ranked by their FDR and selected for plotting.

**PhastCon score and open chromatin signal comparison**

Zebrafish phastCon scores computed based on multiple sequence alignment between 8 vertebrate species (human: hg19, mouse: mm9, X. tropicalis: xenTro2, tetraodon: tetNig2, fugu: fr2, stickleback: gasAcu1, medaka: oryLat2, zebrafish: danRer7) were downloaded from UCSC. DeepTools (version 2.5.0.1)^28^ was used to scale all peaks to the same length and then calculate the phastCon scores of each 10 bp bin for making the aggregate plots. Average phastCon scores were calculated across each entire peak before being used for Wilcoxon test (two-sided).

Signal coverage files of all four ATAC-seq samples were merged together and deepTools (version 2.5.0.1)^28^ was used to plot this overall ATAC-seq signal within a ±3 kb window of ATAC-seq peak midpoints (bin size 5 bp). To statistically test if the open chromatin signals were different, the average signal intensity within ±250 bp of the midpoints was calculated for each ATAC-seq peak and used for Wilcoxon test (two-sided).

**Annotating aCNEs with chromatin states of 127 epigenomes**

Chromatin states that were defined by 5 chromatin marks (H3K4me3, H3K4me1, H3K36me3, H3K27me3, H3K9me3) for 127 human tissues/cell types were retrieved from Roadmap Epigenomics Mapping Consortium^4^ (<http://egg2.wustl.edu/roadmap/web_portal/chr_state_learning.html>). Human aCNEs were intersected with the dense bed files for each of the 127 epigenomes (http://egg2.wustl.edu/roadmap/data/byFileType/chromhmmSegmentations/ChmmModels/coreMarks/jointModel/final/) using BEDtools^23^ (intersectBed, Version 2.19.1) to determine the chromatin state of each aCNE region in different epigenomes. If one aCNE region overlaps multiple chromatin states in one epigenome, the state that shared the longest overlap with the aCNE were attributed to the aCNE. Each chromatin state was represented by an integer ranging from 1 to 15, which was defined by Roadmap Epigenomics Project. Clustering was performed with Manhattan distance and Ward.D clustering methods in R. Heatmaps showing the chromatin state of each human aCNE region were plotted using the same color scheme as the Roadmap Epigenomics Project.

To simplify the analysis, we collapsed the 15 chromatin states into 5 major categories, including Active (1: Active TSS, 2: Flanking Active TSS, 3: Transcr. at gene 5’ and 3, 4: Strong transcription, 5: Weak transcription, 6: Genic enhancers, 7: Enhancers), Bivalent (10: Bivalent/Poised TSS, 11: Flanking Bivalent TSS/Enh, 12: Bivalent Enhancer), Polycomb repressed (13: Repressed Polycomb, 14: Weak Repressed Polycomb), Quiescent (15: Quiescent/Low) and Others (8: ZNF genes & repeats, 9: Heterochromatin). If an aCNE region was associated with an Activestate in more than 80% of the 127 tissues/cell types, the aCNE was considered as constitutively Active. If an aCNE region was Polycomb repressed or Quiescent in more than 70% of the 127 tissues/cell types and Active in at least one epigenome, it was considered as Active in a lineage specific manner.

**Assessing the genomic and epigenomic features of anciently conserved open regions via permutation analysis**

Zebrafish ATAC-seq peaks and human DNase sites were first divided into promoter and non-promoter categories using the same criteria mentioned before (-3 kb to 1 kb of a TSS). Then permutation analysis for total, promoter, and non-promoter aCNEs were done using the matched background set as mentioned below.

|  | aCNEs | Permutation |
| --- | --- | --- |
| Zebrafish aCNEs (total) | 4496 ATAC-seq peaks that were identified as aCNEs conserved with both human and mouse | For each permutation, 4496 regions randomly selected from all ATAC-seq peaks (n=200,937) |
| Zebrafish aCNEs (promoter) | 420 zebrafish aCNEs (conserved with both human and mouse) that overlapped promoter regions | For each permutation, 420 regions randomly selected from promoter ATAC-seq peaks (n= 18,927) |
| Zebrafish aCNEs (non-promoter) | 4076 zebrafish aCNEs (conserved with both human and mouse) that did not overlap promoter regions | For each permutation, 4076 regions randomly selected from non-promoter ATAC-seq peaks (n= 182,010) |
| Human aCNEs (total) | 8866 DHSs that were identified as aCNEs conserved with zebrafish | For each permutation, 8866 regions randomly selected from human DNase master list (n=4,193,929) |
| Human aCNEs (promoter) | 1089 human aCNEs that did not overlap promoter regions | For each permutation, 1089 regions randomly selected from promoter human DNase sites (n=298,023) |
| Human aCNEs (non-promoter) | 7777 human aCNEs that did not overlap promoter regions | For each permutation, 7777 regions randomly selected from non-promoter human DNase sites (3,895,906) |

We randomly subsampled the same number of regions from the whole ATAC-seq peak set or DHS sets 1000 times and then compared their genomic features (GC content, region length, TF binding occupancy, chromatin states etc.) with that of aCNEs. Average GC contents were calculated using BEDtools^23^ (nucBed, Version 2.19.1).

For TF occupancy comparison, the TFBS cluster data from ENCODE was used, which contains the binding sites of 161 human TFs/co-factors in 91 different cell types determined by ChIP-seq experiments. If a region overlaps a record from TFBS cluster data by at least one base pair, it is counted as evidence of binding event at that region. We counted the total number of binding events existing in each set of regions (aCNEs VS randomly selected) for each factor included in TFBS cluster data. We normalized the total number of binding events to the total region lengths and then calculated the enrichment Z-score for each factor.

Z-score = $(x-\mu)/\delta$

x: binding event counts of a factor in aCNEs, normalized by region lengths;

μ: mean of binding event counts of a factor in 1000 times permutation, normalized by region lengths

δ: standard deviation of the binding event counts of a factor in 1000 times permutation.

For chromatin state enrichment analysis, the same strategy was used to determine chromatin states in randomly selected DHS regions. For each epigenome and each set of randomly selected regions, the percentage of regions showing Active, Bivalent, Polycomb repressed, Quiescent or Other states was calculated. The percentage distribution was used for calculating enrichment Z-score.

Z-score = $(x-\mu)/\delta$

x: percentage of regions displaying a certain chromatin state in aCNEs

μ: mean of the percentages of regions displaying a certain chromatin state in 1000 times permutation

δ: standard deviation of the percentages of regions displaying a certain chromatin state in 1000 times permutation.

**Processing and analyzing of published mouse cardiac TF ChIP-seq data**

Mouse cardiac ChIP-seq or ChIP-exo data from the following studies were collected for annotating aCNEs.

| Publication | Factors | Cell types/ Tissues | Data processing |
| --- | --- | --- | --- |
| Laurent et al. 2017 (GSE73368) | HAND2 | E10.25-E10.5 hearts | Uniformly processed |
| Luna-Zurita et al. 2016  (GSE72223) | GATA4, NKX2.5, TBX5 | ESC differentiated cardiac precursors (CPs) and cardiomyocytes (CMs) | Uniformly processed |
| He et al. 2014  (GSE52123) | GATA4 | E12.5 heart ventricles, Adult heart ventricles | Uniformly processed |
| May et al. 2012  (GSE32587) | P300 | Postnatal heart | Use peaks provided by authors |
| He et al. 2011  (GSE21529) | GATA4, NKX2.5, TBX5, MEF2A, SRF, P300 | HL1 cardiomyocytes | Use peaks provided by authors |
| Blow et al. 2010  (GSE22549) | P300 | E11.5 hearts | Use peaks provided by authors |

Data from studies of Laurent et al. 2017, Luna-Zurita et al. 2016 and He et al. 2014 were uniformly processed as below. Raw reads were trimmed with Trimmomatic (TRAILING:3 MINLEN:36) before being aligned to mm9 genome assembly by BWA (version 0.7.8) under aln model^14,19^. Reads with mapping quality score > 30 were kept for downstream analysis with SAMtools (version 1.2) (samtools view -b -q 30). Replicates for the same sample were merged for peak calling by MACS2 (version 2.7.9)^20^ (-q 0.01 -g 1.87e9). Peaks called by MACS2 (version 2.7.9)^20^ (Laurent et al. 2017, Luna-Zurita et al. 2016 and He et al. 2014) or provided by the authors (May et al. 2012, He et al. 2011 and Blow et al. 2010) were intersected with the mouse DHSs conserved with GFP+/- specific ATAC-seq peaks. If a cardiac TF or P300 peak overlaps a mouse DHS by at least one base pairs, this aCNE is considered as bound by this factor.

To generate tracks shown in Supplementary Fig. 1a, H3K27ac^2^ and cardiac transcription factor (GATA4, NKX2.5, TBX5)^1^ ChIP-seq data were used. Alignment bam files (provided by authors) of replicates were merged. Then BEDtools^23^ (genomecov, Version 2.19.1) was used to compute the reads coverage based on the merged bam files for track display.

**Motif analysis for aCNEs**

AME (version 5.0.1)^26^ was used for discovering motifs enriched within each category of the validated heart enhancers. The JASPAR CORE vertebrate non-redundant motif database^29^ was used for this analysis. Motifs with adjusted (Bonferroni correction) p-value < 0.1 were considered as significantly enriched.

To identify GATA motifs in GFP+ and GFP- specific aCNEs, 6 Motifs preferred by GATA family TFs (MA0035.3 Gata1, MA0036.2 GATA2, MA0037.2 GATA3, MA0140.2 GATA1::TAL1, MA0482.1 Gata4 and MA0766.1 GATA5) were extracted from JASPAR core vertebrate motif database^29^ and scanned within the 162 GFP+ specific, human-zebrafish conserved ATAC-seq peaks by FIMO (version 4.12.0)^26^. If an ATAC-seq peak has a stretch of sequence that matches any of the 6 GATA motifs scanned (q < 0.3), this peak was counted as GATA motif positive. If different GATA motifs match to similar regions of a peak, it is only considered as one GATA motif occurrence in this peak due to the information redundancy within different GATA motifs. Only if multiple motifs hit non-overlapping regions of a peak will this peak be counted as having multiple GATA motifs. The 0.3 threshold of q-score was determined by empirical evidence that the GATA motif mutated in experiments described in Supplementary Fig. 6 matches GATA5 motif with a q-value of 0.291 in motif scanning. The full scanning results can be seen in Supplementary Data 5.

**Supplementary references**

1. Luna-Zurita, L. *et al.* Complex Interdependence Regulates Heterotypic Transcription Factor Distribution and Coordinates Cardiogenesis. *Cell* **164,** 999–1014 (2016).

2. Wamstad, J. a *et al.* Dynamic and coordinated epigenetic regulation of developmental transitions in the cardiac lineage. *Cell* **151,** 206–20 (2012).

3. Bogdanovic, O. *et al.* Dynamics of enhancer chromatin signatures mark the transition from pluripotency to cell specification during embryogenesis. *Genome Res.* **22,** 2043–53 (2012).

4. Kundaje, A. *et al.* Integrative analysis of 111 reference human epigenomes. *Nature* **518,** 317–330 (2015).

5. Devine, W. P., Wythe, J. D., George, M., Koshiba-Takeuchi, K. & Bruneau, B. G. Early patterning and specification of cardiac progenitors in gastrulating mesoderm. *Elife* **3,** e03848 (2014).

6. Bessa, J. *et al.* Zebrafish enhancer detection (ZED) vector: a new tool to facilitate transgenesis and the functional analysis of cis-regulatory regions in zebrafish. *Dev. Dyn.* **238,** 2409–17 (2009).

7. Li, Q. *et al.* A systematic approach to identify functional motifs within vertebrate developmental enhancers. *Dev. Biol.* **337,** 484–495 (2010).

8. Kawakami, K. Transposon tools and methods in zebrafish. *Dev. Dyn.* **234,** 244–254 (2005).

9. Kwan, K. M. *et al.* The Tol2kit: a multisite gateway-based construction kit for Tol2 transposon transgenesis constructs. *Dev. Dyn.* **236,** 3088–99 (2007).

10. Zhou, Y. *et al.* Latent TGF-β binding protein 3 identifies a second heart field in zebrafish. *Nature* **474,** 645–8 (2011).

11. Huang, C.-J., Tu, C.-T., Hsiao, C.-D., Hsieh, F.-J. & Tsai, H.-J. Germ-line transmission of a myocardium-specific GFP transgene reveals critical regulatory elements in the cardiac myosin light chain 2 promoter of zebrafish. *Dev. Dyn.* **228,** 30–40 (2003).

12. Kikuchi, K. *et al.* Primary contribution to zebrafish heart regeneration by gata4 + cardiomyocytes. *Nature* **464,** 601–605 (2010).

13. Andrew, S. FastQC: A quality control tool for high throughput sequence data. (2010).

14. Bolger, A. M., Lohse, M. & Usadel, B. Trimmomatic: A flexible trimmer for Illumina sequence data. *Bioinformatics* **30,** 2114–2120 (2014).

15. Dobin, A. *et al.* STAR: Ultrafast universal RNA-seq aligner. *Bioinformatics* **29,** 15–21 (2013).

16. Anders, S., Pyl, P. T. & Huber, W. HTSeq-A Python framework to work with high-throughput sequencing data. *Bioinformatics* **31,** 166–169 (2015).

17. Robinson, M. D., McCarthy, D. J. & Smyth, G. K. edgeR: a Bioconductor package for differential expression analysis of digital gene expression data. *Bioinformatics* **26,** 139–140 (2010).

18. Reimand, J., Kull, M., Peterson, H., Hansen, J. & Vilo, J. G:Profiler-a web-based toolset for functional profiling of gene lists from large-scale experiments. *Nucleic Acids Res.* **35,** (2007).

19. Li, H. & Durbin, R. Fast and accurate short read alignment with Burrows-Wheeler transform. *Bioinformatics* **25,** 1754–1760 (2009).

20. Zhang, Y. *et al.* Model-based Analysis of ChIP-Seq (MACS). *Genome Biol.* **9,** R137 (2008).

21. Stark, R. & Brown, G. DiffBind: differential binding analysis of ChIP-Seq peak data. (2011).

22. Heinz, S. *et al.* Simple Combinations of Lineage-Determining Transcription Factors Prime cis-Regulatory Elements Required for Macrophage and B Cell Identities. *Mol. Cell* **38,** 576–589 (2010).

23. Quinlan, A. R. & Hall, I. M. BEDTools: A flexible suite of utilities for comparing genomic features. *Bioinformatics* **26,** 841–842 (2010).

24. Lex, A., Gehlenborg, N., Strobelt, H., Vuillemot, R. & Pfister, H. UpSet: Visualization of Intersecting Sets. *IEEE Trans. Vis. Comput. Graph.* **20,** 1983–1992 (2014).

25. Heger, A., Webber, C., Goodson, M., Ponting, C. P. & Lunter, G. GAT: a simulation framework for testing the association of genomic intervals. *Bioinformatics* **29,** 2046–8 (2013).

26. Bailey, T. L. *et al.* MEME SUITE: tools for motif discovery and searching. *Nucleic Acids Res.* **37,** W202–W208 (2009).

27. McLean, C. Y. *et al.* GREAT improves functional interpretation of cis-regulatory regions. *Nat. Biotechnol.* **28,** 495–501 (2010).

28. Ramírez, F. *et al.* deepTools2: a next generation web server for deep-sequencing data analysis. *Nucleic Acids Res.* **44,** W160–W165 (2016).

29. Mathelier, A. *et al.* JASPAR 2016: A major expansion and update of the open-access database of transcription factor binding profiles. *Nucleic Acids Res.* **44,** D110–D115 (2016).
