# Supplementary material for: Heart enhancers with deeply conserved regulatory activity are established early in zebrafish development
Source: Nat Commun. 2018 Nov 26;9:4977. doi: 10.1038/s41467-018-07451-z (PMC6255839; doi:10.1038/s41467-018-07451-z)
Supplement: Supplementary file 3 — Description of Additional Supplementary Files [file 41467_2018_7451_MOESM3_ESM.pdf]

Description of Additional Supplementary Files

Supplementary Data 1: genomic coordinates of GFP+ specific, GFP- specific and shared ATAC-seq peaks

Supplementary Data 2: Metadata of all zebrafish aCNEs (with human and/or mouse). Each row represents a zebrafish aCNE. Explanation of specific columns are listed below

- **Zv9_CNE_human**: Zv9 genome coordinates of the CNEs that overlap this ATAC-seq peak. The CNEs are conserved between zebrafish and human. Multiple CNEs mapped to the same ATAC-seq peaks were separated by comma. "NA" indicates this zebrafish aCNE was not identified as zebrafish-human aCNEs (but was zebrafish-mouse aCNEs).

- **hg19_alignment_type**: whether these zebrafish-human CNEs were identified through direct or indirect alignment. In cases of multiple CNEs, alignment types are listed in the same order as that in the "Zv9_CNE_human" column.

    - direct: direct alignment approach used in the zCNE database (Hiller et al, 2013)
    - indi_trans: transitivity approach (indirect) used in the zCNEs database (Hiller et al, 2013)
    - indi_recons: ancestry reconstruction approach (indirect) used in the zCNE database (Hiller et al, 2013)
    - indi_garCNE: transitivity approach (indirect) used in spotted gar genome paper (Braasch et al, 2016)
- **hg19_overlap**: how many base pairs the zebrafish aCNE overlap the zebrafish-human CNE(s). In cases of multiple CNEs, overlapping base pairs are listed in the same order as that in the "Zv9_CNE_mouse" column.

- **hg19_DHS**: coordinates of the orthologous human aCNEs in hg19 genome assembly.

- **hg19_VistaEnhancer**: whether this epigenetically conserved region (human DHSs) overlaps human genomic elements that have been included in the VISTA enhancer browser (https://enhancer.lbl.gov/). "none" means no overlap, "NA" indicates this aCNE region is not conserved with human. For aCNE regions that overlap VISTA enhancers, records are formatted as "VISTA element ID_enhancer assay results_total overlapping base pairs".

- **hg19_UltraConserved**: whether the orthologous human regions of this aCNE overlaps any ultraconserved elements (Bejerano et al, 2004). "none" means no overlap, "NA" indicates that this aCNE is not zebraish-human aCNE. For aCNE regions that overlap, records are formatted as "ultraconserved element ID_total overlapping base pairs".

- **hg19_DHS_merge**: coordinates of the orthologous human aCNEs in hg19 genome assembly, after those within 200 bp distance were merged.

For the equivalent mouse columns (Zv9_CNE_mouse, mm9_alignment_type, mm9_overlap, hg19_DHS, mm9_VistaEnhancer, mm9_DHS_merge), the same formats were used as above.

Within the “aCNE GFP+ specific ATAC” tab, ChIP-seq or ChIP-exo data from 6 studies were intersected with the aCNE regions (>=1 bp overlap required) and the results were recorded as 0 (no overlap between the TF binding site aCNE regions) or 1 (TF binding site overlaps the aCNE region).

Supplementary Data 3: Mouse cardiac TF and P300 ChIP-seq data collected from previous publication for annotating aCNEs**.**

Supplementary Data 4: In vivo enhancer assays and transgenic lines generated in this study

Supplementary Data 5: Full outputs of GATA motif scanning in GFP+ or GFP- specific aCNE regions using FIMO

Supplementary Data 6: mRNA-seq and ATAC-seq metrics
